# Locus Coeruleus Noradrenergic‐Spinal Projections Contribute to Electroacupuncture‐Mediated Antinociception in Postoperative Pain in Mice

**DOI:** 10.1002/advs.202501182

**Published:** 2025-05-19

**Authors:** Wen‐Guang Chu, Ru Zhang, Hai‐Tao Li, Ying‐Chun Li, Hui Ding, Zhen‐Zhen Li, Wen‐Juan Han, Fei Wang, Xing‐Xing Zheng, Hong‐Hui Mao, Hua Yuan, Sheng‐Xi Wu, Rou‐Gang Xie, Ceng Luo

**Affiliations:** ^1^ Department of Neurobiology School of Basic Medicine Fourth Military Medical University Xi'an 710032 China; ^2^ Department of Anesthesiology Tangdu Hospital Fourth Military Medical University Xi'an 710016 China; ^3^ School of Medicine Yanan University Yan'an 716000 China; ^4^ The Fourteenth Squadron of the Fourth Regiment School of Basic Medicine Fourth Military Medical University Xi'an 710032 China; ^5^ College of Life Sciences Northwest University Xi'an 710069 China; ^6^ Department of Rehabilitation Medicine Xijing Hospital Fourth Military Medical University Xi'an 710032 China; ^7^ Innovation Research Institute Xijing Hospital Fourth Military Medical University Xi'an 710032 China

**Keywords:** electroacupuncture, locus coeruleus, noradrenaline, postoperative pain, ST36

## Abstract

Postoperative pain remains a significant challenge in healthcare. Electroacupuncture (EA) has gained polarity in helping manage surgical pain and showed beneficial effects on enhancing postoperative analgesia, decreasing opioid requirement. Despite this, the precise mechanisms underlying these actions are poorly understood. Evidence shows the involvement of noradrenaline (NE) in the action of EA. However, the precise identity of the NE source after EA treatment, its mechanisms of action, and the circuitry locus in the pain‐regulating pathway remain elusive. It is shown that plantar incision (PI) leads to hypoactivity of noradrenergic neurons in the locus coeruleus (LC), which brings about impaired NE release in the spinal dorsal horn (SDH). EA treatment normalizes the abnormal hypoexcitability of LC noradrenergic neurons after PI and thus triggers enhanced NE release in the SDH. Optogenetic inhibition of LC noradrenergic neurons eliminates EA‐induced NE release and antinociceptive effects after PI, while activation of these neurons mimics EA‐induced NE release and antinociception. The resultant increased NE release after EA activates spinal α_2A_‐adrenoceptor and inhibits CaMKII signaling, which in turn depresses spinal excitatory neuronal hyperexcitability and eventually relieves postoperative pain. It is concluded that LC noradrenergic‐spinal projections and subsequent α_2A_‐adrenoceptor–CaMKII signaling cascades in the SDH contribute to EA‐induced antinociception in postoperative pain.

## Graphical abstract

How locus coeruleus noradrenergic‐spinal projections and subsequent α_2A_ adrenoceptors‐CaMKII signaling cascades contribute to electroacupuncture‐induced antinociception in postoperative pain in mice is described.

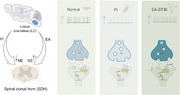

## 1 Introduction

Postoperative pain represents a common clinical issue. It is reported to occur in ∼80% of patients undergoing various common surgeries.^[^ ^1^, ^2^, ^3^ ^]^ Inadequate pain management frequently leads to a broad range of negative consequences, including the development of chronic pain, increased opioid intake, prolonged healing process, etc.^[^ ^3^, ^4^ ^]^ Thus, clinical exploitation of novel non‐opioid‐based treatment options targeting pathway‐specific mechanisms relevant for postoperative pain is urgently needed.

Acupuncture has been widely used for pain treatment in clinical practice for more than 3000 years. Although it originated in China, acupuncture has now gained popularity as an integrated pain management approach worldwide.^[^ ^5^ ^]^ For example, accumulating evidence from clinical trials and experimental studies has documented the potent efficacy of acupuncture in alleviating multiple types of pain, spanning from acute to chronic pain, including postoperative pain.^[^ ^3^, ^6^, ^7^, ^8^ ^]^ Given the precision and standardization of stimulation frequency, intensity, and duration, electroacupuncture (EA), one of the modalities of acupuncture, has been widely employed in pre‐, peri‐, and post‐operative pain control and provided beneficial analgesic effects with the least side effects.^[^ ^9^, ^10^ ^]^ Despite these advances, the precise mechanism underlying EA‐induced analgesia in postoperative pain is far from being understood, especially in the brain.

Over the last several decades, emerging studies have demonstrated the involvement of several neurochemicals in the action of EA, including noradrenaline (NE), by using diverse animal models.^[^ ^10^, ^11^, ^12^, ^13^, ^14^ ^]^ However, the precise identity of NE source after EA treatment, their mechanisms of action, and their circuitry locus in the pain‐regulating pathway have remained elusive. The locus coeruleus (LC) is known to contain the largest group of noradrenergic neurons in the central nervous system (CNS), thus providing the principal supply of NE to the brain and spinal cord and thereby modulating an array of brain functions, including pain sensitization.^[^ ^15^ ^]^ However, it remains controversial and debated regarding the LC as the main source of NE action after EA treatment. For example, some studies reported that EA increases LC activity,^[^ ^16^, ^17^, ^18^, ^19^, ^20^ ^]^ whereas others demonstrated that EA inhibits LC neuronal excitability.^[^ ^21^, ^22^, ^23^, ^24^ ^]^ Thus, we are interested in asking the questions: What plastic changes would LC noradrenergic neurons undergo after surgical injury? How does EA modulate these abnormal plastic changes of LC noradrenergic neurons to alleviate pain hypersensitivity? Which cellular processes underlie these actions?

The spinal dorsal horn (SDH) is the first relay station of pain signal processing from the periphery to the CNS. Nociceptive sensory flow in the spinal cord often undergoes descending modulation from supraspinal regions such as the LC, which is crucial in pain regulation.^[^ ^25^, ^26^, ^27^, ^28^ ^]^ Given the importance of LC noradrenergic‐spinal projections in the descending pain control, it is fascinating to elucidate how EA exerts top‐down regulation of spinal nociceptive inputs via regulation of LC noradrenergic activity to relieve postoperative pain.

By combining behavioral surveys, electrophysiology, optogenetics, fiber photometry recording, miniscope GRIN lens calcium imaging, as well as biochemical assays, we have now depicted the cellular and circuit basis for the action of EA in the processing of postoperative pain. Following the PI challenge, the activity of LC noradrenergic neurons gets downregulated, which brings about the impaired release of NE in the SDH. Delivery of EA treatment restores the dysregulated hypoexcitability of LC noradrenergic neurons and thus triggers enhanced release of NE in the SDH. The resultant increased NE release in the SDH activates α_2A_‐adrenoceptor and inhibits CaMKII signaling, which in turn depresses spinal excitatory neuronal hyperexcitability and eventually relieves postoperative pain. We believe this study sheds new light on the functional capability of LC noradrenergic‐spinal projections in the EA‐induced analgesia in postoperative pain.

## 2 Results

### 2.1 EA Alleviates Mechanical Allodynia and Thermal Hyperalgesia Induced by PI

**Figure 1:**
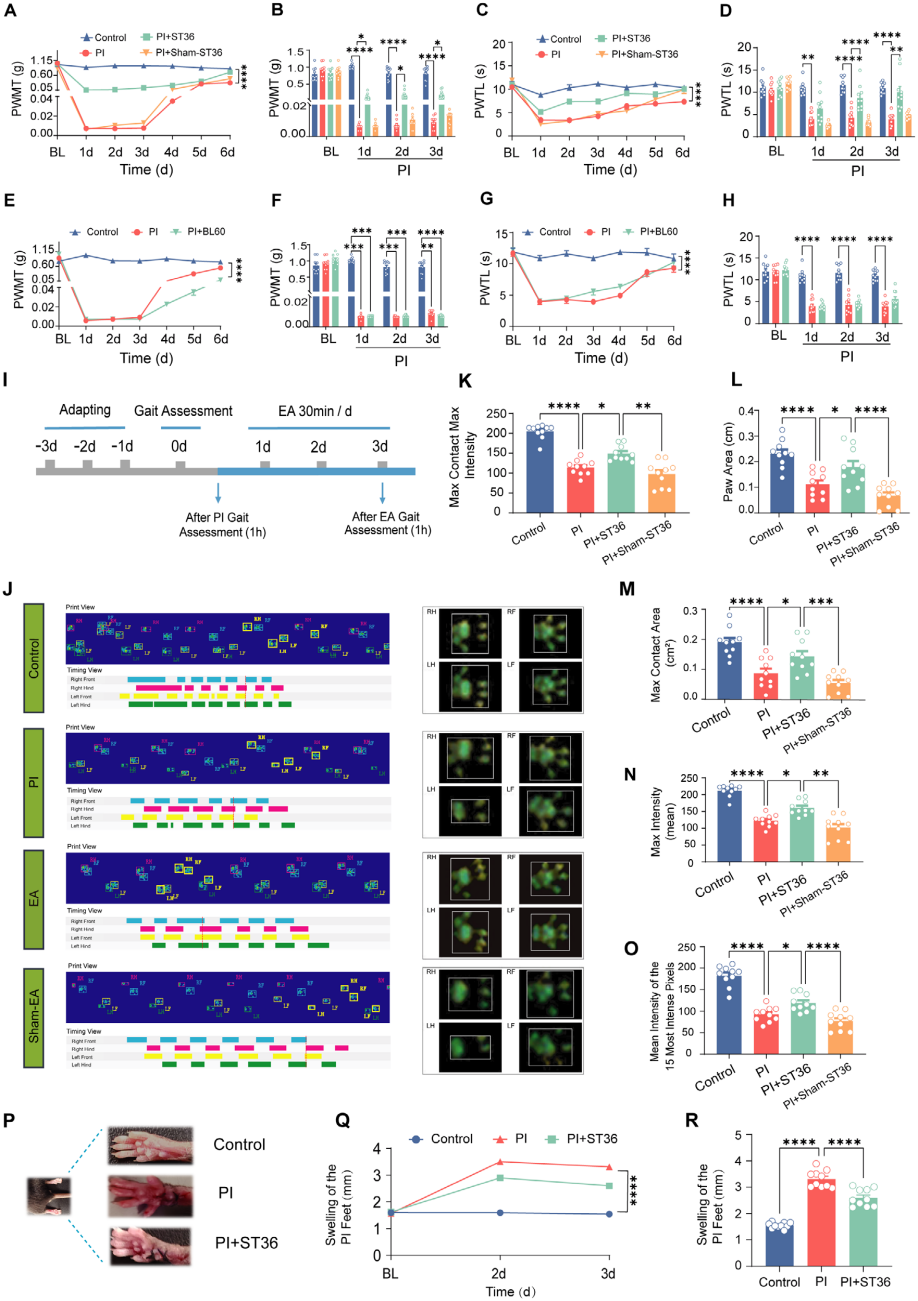
EA alleviates mechanical allodynia and thermal hyperalgesia induced by PI. A,C) The time course showing the effects of consecutive EA administration at ST36 acupoint for 6 days on mechanical and thermal pain hypersensitivity in the control group, PI group, PI+ST36 group, and sham‐EA group (per group, *n* = 10). ****p < 0.0001 by Friedman's M test. B,D) Magnitude of mechanical threshold to von Frey hairs (B) and thermal latency to radiant heat stimuli (D) in the above groups. *p < 0.05, **p < 0.01, ****p < 0.0001 by Friedman's M test and Dunn's multiple comparisons test. E,G) The time course showing the effects of consecutive EA administration at BL60 acupoint for 6 days on mechanical and thermal pain hypersensitivity in control group, PI group, and PI +BL60 group (per group, *n* = 10). ****p < 0.0001 by Friedman's M test (E) and Repeated Measures ANOVA test (G). F,H) Magnitude and time course of mechanical threshold to von Frey hairs (F) and thermal latency to radiant heat stimuli (H) in the above groups. **p < 0.01, ***p < 0.001, and ****p < 0.0001 by Friedman's M test and Repeated Measures ANOVA test. I) Experimental schematic diagram for Gait assessment. J) Representative Catwalk gait, including Print view, Timing view, Interactive Footprint Measurements, and 3D Footprint Intensities. K–O) Gait parameters: (K) Max Contact Area, (L) Paw Area, (M) Max Contact Max Intensity, (N) Max Intensity, and (O) Mean Intensity of the 15 Most Intense Pixels. Statistical changes of gait parameters between the control group, PI group, EA group, and Sham‐EA group (per group, *n* = 10). *p < 0.05, **p < 0.01, ***p < 0.001, and ****p < 0.0001 by Kruskal–Wallis H test and One‐way ANOVA. P) Images of mice paw swelling in the control group, PI group, and EA group. Q) The swelling of the plantar incision‐induced (PI) feet in millimeters before the plantar incision and on the second and third days after the incision. ****p < 0.0001 by Friedman's M test. (R) The swelling of the hindpaw in control group, PI group, EA group on 3d post‐PI. **p < 0.01, ****p < 0.0001 by the Kruskal–Wallis H test with Dunn's multiple comparisons test. Data are represented as mean ± S.E.M. See Table S2(Supporting Information) for detailed statistical information. PWMT, paw withdrawal mechanical threshold; PWTL, paw withdrawal thermal latency; BL, Basal.

To elucidate the role of EA in postoperative pain, we employed a well‐accepted mouse model of PI in mice (schematic diagram shown in Figure S1A, Supporting Information).^[^ ^29^ ^]^ Following unilateral PI, mice developed mechanical allodynia and thermal hyperalgesia in ipsilateral hindpaw, as characterized by a leftward and upward shift in the stimulus–response curve over basal curve, reduced mechanical threshold in response to von Frey hairs as well as shortened thermal latency to radiant heat stimulation applied to plantar surface of injured hindpaw (Figure S1B–D, Supporting Information). This mechanical and thermal pain hypersensitivity peaked at 1d post‐incision, persisting at a plateau for 3 days and then gradually returning to basal level at 6d post‐incision (Figure S1C,D, Supporting Information), which nicely reflected the pain time course endured by clinical surgical patients. Zusanli acupoint (ST36) has been associated with pain treatment in clinical trials.^[^ ^30^, ^31^ ^]^ In PI‐treated mice, we observed that daily delivery of EA with 2 Hz/100 Hz alternating frequency at 2 mA intensity for 30 min over consecutive 6 days at bilateral ST36 acupoint significantly improved mechanical allodynia and thermal hyperalgesia, manifesting as elimination of reduction of mechanical threshold and thermal latency after PI challenge (**Figures** **1**A‐D and S1E, Supporting Information). By contrast, sham EA treatment at ST36 acupoint did not produce this antinociceptive effect (Figure 1A–D and Figure S1E, Supporting Information). In further support of EA‐induced analgesia in postoperative pain, we tested the effect of another widely used acupoint for analgesia in the clinic, BL60. Unexpectedly, we did not observe the obvious change in the mechanical and thermal pain hypersensitivity after EA stimulation at BL60 acupoint in PI‐treated mice (Figure 1E–H and Figure S1F, Supporting Information). This might be due to the fact that BL60 acupoint is located very close to the injury site. Thus, we chose ST36 acupoint for further mechanistic analysis.

It is well known that pain is a significant factor influencing the walking gait of mice. The antinociceptive action by EA in the postoperative pain was further strengthened by the gait assessment using CatWalk paradigm (schematic diagram shown in Figure 1I). As shown in Figure 1J–O, PI caused obvious gait disturbances in mice. Combined analysis of print view, timing view, interactive footprint measurements, and footprint intensity revealed that PI‐injured mice exhibited abnormal gait parameters, including reduced max contact intensity, paw area, and max contact area in the injured hindpaw (Figure 1J–O). This abnormality in gait parameters was ameliorated by EA stimulation at ST36 acupoint but not by sham‐EA treatment (Figure 1J–O). In addition to pain intensity, PI induced a marked swelling of the injured hindpaw as well, which was significantly improved after EA treatment (Figure 1P–R). Taken together, these results indicate that EA produces a potent antinociceptive effect in postoperative pain in mice.

### 2.2 EA Activates Noradrenergic Neurons in the LC

**Figure 2:**
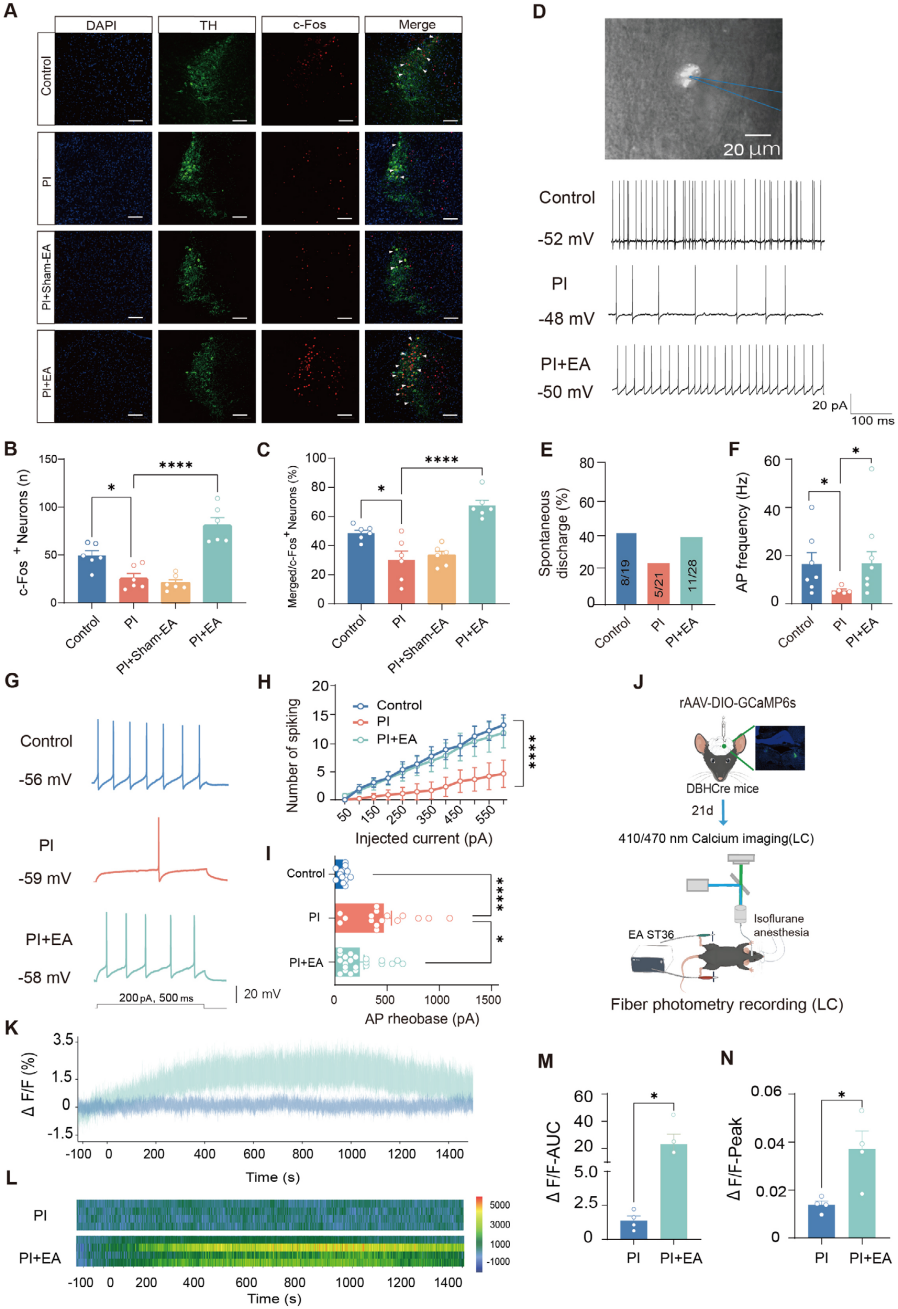
EA activates noradrenergic neurons in the LC. A–C) Representative immunofluorescence images (A) and quantitative summary (B,C) showing PI induced a reduction of c‐Fos expression in TH‐positive neurons in the LC and EA treatment reversed this reduction (per group, *n* = 3 mice, 6 slices/group). Scale bars: 50 µm. *p < 0.05, ****p < 0.0001 by one‐way ANOVA followed by Tukey's HSD (Honestly Significant Difference) post‐hoc analyses. D) Whole‐cell patch‐clamp recordings in LC brain slices from DBH‐Cre mice with infusion of AAV2/9‐DIO‐mCherry in the LC to label LC noradrenergic neurons. Typical traces of spontaneous firing of LC noradrenergic neurons in different groups are shown. Scale bar: 20 µm. E,F) Quantitative summary showing the incidence rate of spontaneous firing (E) and spontaneous firing frequency (F) was significantly reduced at 1d after PI injury and reversed after EA treatment (*n* = 5–8). *p < 0.05 by the Kruskal–Wallis H test followed by Dunn's multiple comparisons test HSD (Honestly Significant Difference) post‐hoc analyses. G,H) Representative traces of action potentials (AP) (G) and I–O curve (H) in response to a depolarizing current step showing reduced AP frequency after PI injury and restoration after EA treatment (*n* = 9–18). ****p < 0.0001, by Friedman's M test. I) A higher rheobase was observed after PI and reversed by EA stimulation (*n* = 13–22). *p < 0.05, ****p < 0.0001 by the Kruskal–Wallis H test followed by Dunn's multiple comparisons test HSD. J) Experimental schematic diagram showing fiber photometry recording in the LC. K,L) Typical examples of traces (K) and heat maps (L) of Ca^2+^ transients in LC noradrenergic neurons induced by EA treatment for 25 min. M,N) Quantitative summary of Ca^2+^ transients in both peak amplitude (M) and area under curve (AUV) (N) prior to and after EA treatment (per group, *n* = 4). *p < 0.05, by two‐tailed unpaired *t*‐test. Data are represented as mean ± S.E.M. See Table S2 (Supporting Information) for detailed statistical information.

Then, how does EA accomplish analgesia in postoperative pain? We first performed an unbiased whole‐brain screening for the activation region by EA delivery using a neuronal activity marker, immediate early gene product c‐Fos. Immunofluorescence staining demonstrated that EA delivery leads to an increase of c‐Fos immunoreactivity in many brain regions as compared to sham‐EA treatment (Figure S2A,B, Supporting Information), including primary and secondary motor cortex (M1, M2), visual cortex (V1), amygdala, thalamus, hypothalamus, periaqueductal (PAG), LC, and rostral ventromedial medulla (RVM). Amongst which, LC brought to our attention, since LC is assumed to be a crucial area involved in descending pain modulation.^[^ ^26^, ^28^ ^]^ We thus sought to examine the changes of LC neuronal activity after PI injury and EA treatment by employing several different approaches. Following PI 1d injury, the number of Fos‐immunoreactive neurons in the LC was markedly reduced (**Figure** **2**A,B), indicating a reduced LC neuronal activity under the postoperative pain state. This reduced LC activity was robustly reversed by EA stimulation, as visualized by restoration of c‐Fos immunoreactivity, but was not affected by sham‐EA treatment (Figure 2A,B). LC is known to be rich in tyrosine hydroxylase (TH)‐positive neurons, which release noradrenaline to regulate a variety of brain functions.^[^ ^32^, ^33^, ^34^ ^]^ Further analysis of dual immunofluorescence with c‐Fos and TH revealed that EA treatment is able to normalize the reduced activity of TH‐positive neurons after PI injury (Figure 2A,C).

To further characterize the functional changes of LC noradrenergic neurons after PI injury and EA treatment, we performed whole‐cell patch‐clamp recordings in LC brain slices from DBH‐Cre mice with infusion of AAV2/9‐DIO‐mCherry in the LC to label LC noradrenergic neurons. In the control state, 42% (8 out of 19 neurons) of LC noradrenergic neurons exhibited spontaneous firing (Figure 2D,E). The incidence rate of spontaneous firing was significantly reduced at 1d after PI injury, which was reversed by EA treatment (Figure 2D,E). Analysis of spontaneous firing frequency revealed that PI‐injured LC noradrenergic neurons display much lowered firing frequency, while EA stimulation excludes the PI‐induced reduction in spontaneous firing frequency (Figure 2D,F). These results provide a hint that LC noradrenergic neurons may become less active under the postoperative pain state, and EA stimulation can reactivate them. In support of this assumption, we further tested the intrinsic excitability of LC noradrenergic neurons without spontaneous firing derived from control, PI‐injured, as well as PI‐injured mice receiving EA stimulation. Following PI injury, we found that the excitability of LC noradrenergic neurons was profoundly depressed at 1d post‐PI (Figure 2G). A detailed input (current intensity)–output (action potential [AP] frequency) curve in response to a depolarizing current step was drawn for each group of mice (Figure 2H). PI‐injured LC noradrenergic neurons displayed a significant reduction in firing frequency, as characterized by a downward shift of the input–output (I–O) curve over the control curve (Figure 2H). This deviation in the I–O curve after PI injury was normalized by EA treatment (Figure 2H). Meanwhile, a much heightened rheobase was observed in PI‐injured LC noradrenergic neurons as compared to control neurons, and EA stimulation reversed this elevation of rheobase (Figure 2I). Analysis of other AP parameters revealed that AP threshold is reduced after PI injury and restored back to normal after EA delivery, while AP amplitude, AP half‐width, as well as resting membrane potential, were not different in control, PI, and PI plus EA group (Figure S2C–F, Supporting Information). These results suggest that EA produces an antinociceptive effect probably via reversing the abnormal hypoexcitable state of LC noradrenergic neurons caused by PI injury.

To further verify the activation of LC noradrenergic neurons after EA treatment, we performed in vivo real‐time Ca^2+^ photometry recording in the LC. The activity of LC noradrenergic neurons was monitored by stereotaxically injecting an adeno‐associated virus (AAV2/9) carrying DIO‐GCaMP6s into the LC of DBH‐Cre mice and measuring changes in GCaMP6s fluorescence in response to EA stimulation in PI‐injured mice (schematic diagram shown in Figure 2J). Aligning the GCaMP signals with video‐recorded stimulus application revealed that EA stimulation for 25 min induces sustained elevation of Ca^2+^ level, as characterized by marked photometric Ca^2+^ signals in LC noradrenergic neurons (Figure 2K–N), indicating the activation of LC noradrenergic neurons by EA treatment. Overall, we can infer from the above data that EA might accomplish analgesia in postoperative pain via counteracting the impaired function of the LC noradrenergic system associated with PI.

### 2.3 Optogenetic Inhibition of LC Noradrenergic Neurons Excludes EA‐Induced Antinociception

**Figure 3:**
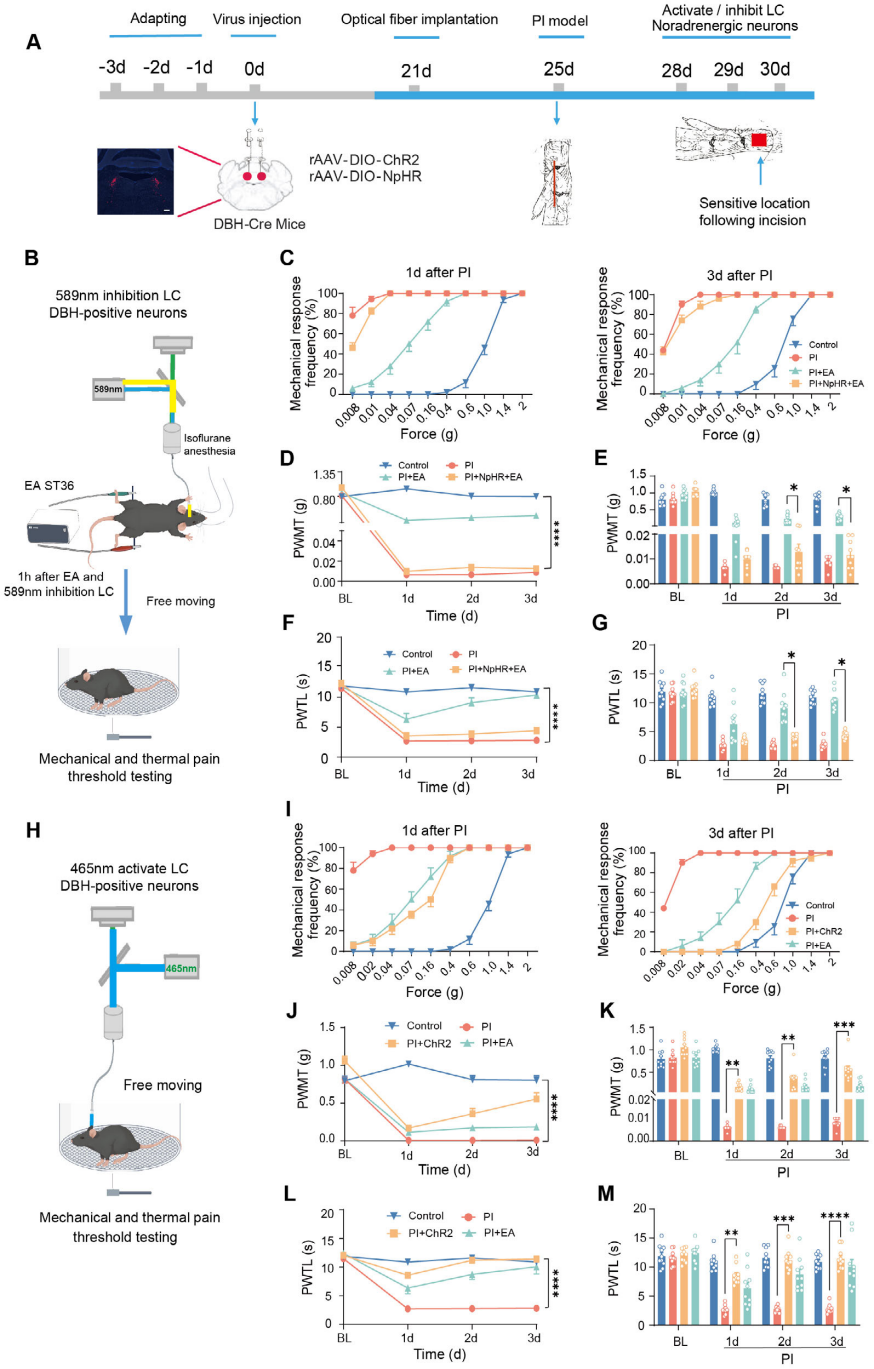
Optogenetic inhibition of LC noradrenergic neurons excludes EA‐induced pain relief. A) Schematic diagram for exploring the role of LC noradrenergic neurons in the EA‐induced analgesia in the postoperative pain. B–G) Schematic diagram (B), stimulus–response curve (C), mechanical threshold (D,E) and thermal latency (F,G) analysis showing optogenetic inhibition of LC noradrenergic neurons excludes EA‐induced inhibition of mechanical allodynia (C–E) and thermal hyperalgesia (F,G) in PI model (per group, *n* = 10 mice). ****p < 0.0001 by Friedman's M test (D,F), *p < 0.05 by Friedman's M test followed by Dunn's multiple comparisons test HSD (E,G). (H–M) Schematic diagram (H), stimulus–response curve (I), mechanical threshold (J,K) and thermal latency (L,M) analysis showing optogenetic activation of LC noradrenergic neurons inhibits PI‐induced mechanical allodynia (I‐K) and thermal hyperalgesia (F,G) (per group, *n* = 10 mice). ****p < 0.0001 by Friedman's M test (J, L), **p < 0.01, ***p < 0.001, ****p < 0.0001 by Friedman's M test followed by Dunn's multiple comparisons test HSD (K, M). Data are represented as mean ± S.E.M. See Table S2 (Supporting Information) for detailed statistical information.

Given the above observed activation of LC noradrenergic neurons by EA stimulation, we went on to ask whether LC noradrenergic neurons are required for EA‐induced antinociception in postoperative pain. To this end, optical approaches were employed for temporally controlled and reversible manipulation of LC noradrenergic neurons by LC infusion of AAV2/9‐DIO‐NpHR (or ChR2) in DBH‐Cre mice (schematic diagram shown in **Figure** **3**A,B). We demonstrated that bilateral optical inhibition of LC noradrenergic neurons largely attenuated the inhibitory effect of EA on PI‐induced mechanical allodynia and thermal hyperalgesia, as visualized by the leftward shift of the stimulus–response curve, reduced mechanical threshold, and shortened thermal latency as compared to the EA group (Figure 3C–G). On the other hand, optical activation of LC noradrenergic neurons itself in PI‐injured mice showed strong ability to suppress the mechanical allodynia and thermal hyperalgesia (Figure 3H‐M), which mimics the antinociceptive effect achieved by EA treatment. The leftward and upward shift of the deviation of the stimulus‐–response curve, as well as reduced mechanical threshold in response to von Frey hairs under the PI‐injured state, was rectified after optical activation of LC noradrenergic neurons (Figure 3I–K). In parallel, PI‐induced reduction of thermal latency to radiant heat stimulation was eliminated as well (Figure 3L,M). In contrast, the above effects were not observed in the control virus group without carrying NpHR or ChR2 (Figure S3A–L, Supporting Information). Taken together, these observations led us to infer that EA‐induced analgesia in postoperative pain is dependent on its activation of LC noradrenergic neurons.

### 2.4 EA Stimulation Leads to NE Release from LC Noradrenergic Neurons in the SDH

**Figure 4:**
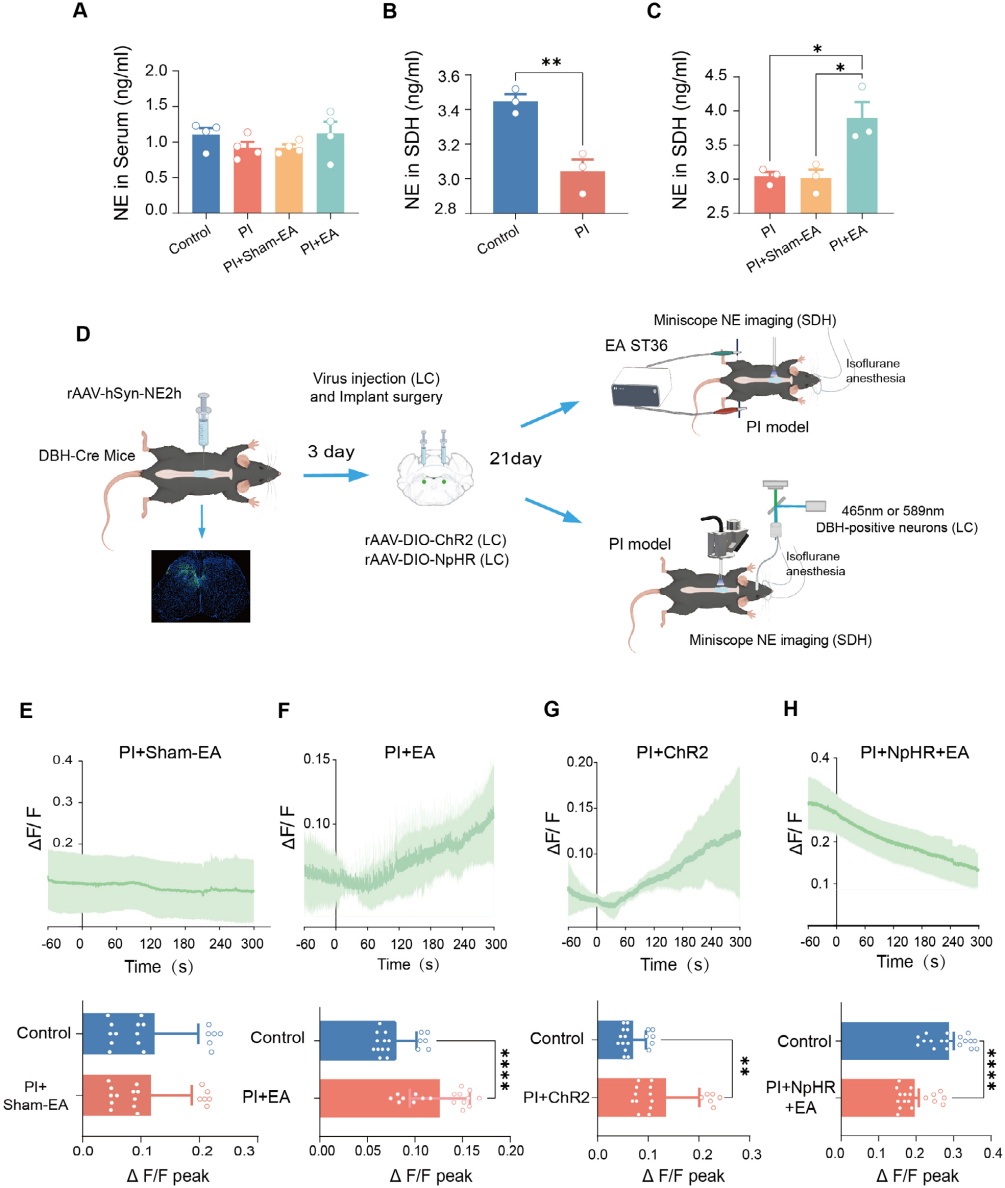
EA stimulation leads to NE release from LC noradrenergic neurons in the SDH. A–C) ELISA assay showing the NE level in the serum (A) and SDH (B,C) in control, PI‐injured mice, and PI‐injured mice receiving EA and sham‐EA treatment (per group, *n* = 3–4 samples of 6–8 mice). **p < 0.01 by two‐tailed unpaired *t*‐test in (B), *p < 0.05 by One‐way ANOVA followed by Tukey's multiple comparisons test HSD (C). D) Schematic illustration depicting the experimental design for monitoring NE release using miniscope recording in the SDH in response to optical stimulation of LC noradrenergic neurons. E–H) Typical traces (upper panels) and quantitative summary (lower panels) showing NE release after sham‐EA (E), EA (F), optogenetic activation of LC noradrenergic neurons (G), and optogenetic inhibition of LC noradrenergic neurons plus EA treatment (H). *n* = 18 trials from 3 mice for each condition. **p < 0.01, ****p < 0.01 by Mann–Whitney U test. Data are represented as mean ± S.E.M. See Table S2 (Supporting Information) for detailed statistical information.

Then, by which mechanism do LC noradrenergic neurons participate in the EA‐induced analgesia? The LC noradrenergic system is the main source of NE in the CNS and is intensively involved in modulating pain and stress‐related disorders and in their comorbidity.^[^ ^33^ ^]^ We next asked whether EA produces pain relief via modulation of NE release from LC noradrenergic neurons. ELISA assay revealed that serum NE level was not altered by PI injury and EA treatment (**Figure** **4**A). However, detailed analysis of NE level in the SDH showed that a much lower level of NE was observed at 1d after PI injury as compared to the control group (Figure 4B). This reduced NE level in the SDH was normalized by EA treatment, but not by sham‐EA treatment (Figure 4C). To further identify whether EA‐induced NE release in the SDH is from LC noradrenergic neurons, we injected AAV2/9‐hsyn‐NE2h into the SDH of DBH‐Cre mice to monitor NE release from LC noradrenergic neurons through miniature fluorescent microscope (miniscope) GRadient INdex (GRIN) lens systems mounted in the lumbar spinal cord (Figure 4D). Meanwhile, Cre‐dependent AAV2/9 carrying ChR2 or NpHR was infused into the LC of DBH‐Cre mice for manipulation of LC noradrenergic neurons (Figure 4D). As shown in Figure 4E,F, EA stimulation elicited a dramatic release of NE in the SDH of PI‐injured mice, manifesting as a gradual increase of fluorescence, while sham‐EA did not (Figure 4E,F; Videos S1 and S2, Supporting Information). This EA‐induced NE release was comparable to that induced by optical activation of LC noradrenergic neurons (Figure 4G; Video S3, Supporting Information). More importantly, optical inhibition of LC noradrenergic neurons eliminated the EA‐induced NE release, and even further inhibited it below the basal level in the SDH (Figure 4H; Video S4, Supporting Information). Altogether, these results suggest that EA might exert antinociceptive effects by activating LC noradrenergic neurons to release NE in the SDH.

### 2.5 EA Treatment Eliminates the Abnormal Potentiation of Spinal Nociceptive Responses after PI Injury

**Figure 5:**
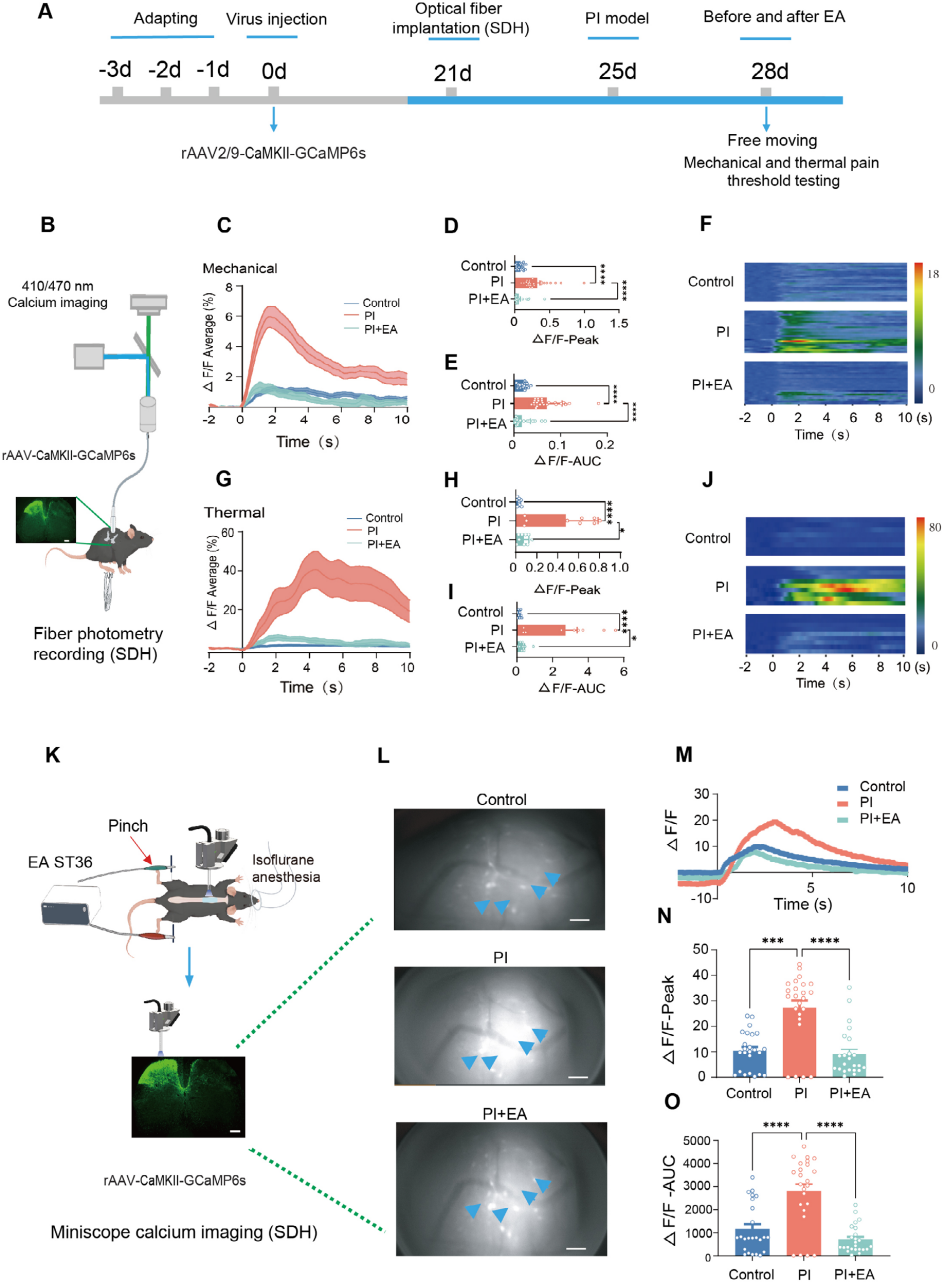
EA treatment eliminates the abnormal potentiation of spinal nociceptive responses after PI injury. A) Schematic diagram for exploring the role of EA stimulation in modulation of spinal nociceptive responses in PI‐injured mice using in vivo real‐time spinal fiber photometry recording B) and GRIN lens miniscope recording K). B–F) Schematic diagram of spinal fiber photometry recording in freely moving mice (B), typical traces (C), quantitative summary of peak amplitude and area under curve (AUC) (D,E,H,I) and heat map F,J) showing Ca^2+^ transients in spinal excitatory neurons induced by mechanical (C‐F) and heat stimulation (G‐J) of the injured hindpaw in control, PI‐injured and PI injury plus EA condition. *n* = 24 trials from 3 mice per group. *p < 0.05, ****p < 0.0001, by the Kruskal–Wallis H test followed by Dunn's multiple comparisons test HSD (D,E,H,I). K–O) Schematic diagram of GRIN lens miniscope imaging in the SDH (K), typical examples of images (L), representative traces (M), quantitative summary of peak amplitude (N) and area under curve (AUC) (O) showing Ca^2+^ transients in spinal excitatory neurons induced by mechanical stimulation of the injured hindpaw in control, PI‐injured and PI injury plus EA condition. *n* = 32 cells from 4 mice per group. ***p < 0.001, ****p < 0.0001 by Kruskal – Wallis H test followed by followed by Dunn's multiple comparisons test HSD (N, O). Data are represented as mean ± S.E.M. See Table S2 (Supporting Information) for detailed statistical information.

SDH is well assumed to be the first gate transmitting nociceptive pain signals from the periphery to the CNS. Nociceptive sensory inflow in the SDH often undergoes descending modulation from supraspinal regions, including LC noradrenergic projections, which is crucial in pain processing.^[^ ^26^, ^28^ ^]^ After getting to know, EA increases NE release from LC noradrenergic projections, we then turned to elucidate how EA regulates spinal nociceptive inputs via these top‐down projections from LC noradrenergic neurons. To this end, we employed in vivo real‐time spinal Ca^2+^ photometry recording and miniscope GRIN lens spinal Ca^2+^ recording at populated neurons and single neuron levels, respectively (schematic diagram shown in **Figure** **5**A). The activity of spinal excitatory neurons was monitored by infusion of AAV2/9‐CaMKII‐GCaMP6s vectors into the lumbar SDH and measurement of changes in GCaMP6s fluorescence in response to mechanical and thermal stimulation applied to the cutaneous receptive field in control mice and PI‐injured mice receiving EA or sham‐EA treatment (Figure 5B–J; Videos S5–S8, Supporting Information). Aligning photometric Ca^2+^ signals in populated spinal excitatory neurons with video‐recorded stimulus application revealed that upon PI injury, a robust increase of Ca^2+^ transients in both the peak amplitude and area of curve (AUC) was elicited by application of mechanical pinch and radiant heat stimulation to the cutaneous receptive field (Figure 5C–J), indicating a facilitating effect of PI injury on spinal excitatory neurons. Interestingly, this potentiated Ca^2+^ response induced by PI injury was greatly attenuated in both the peak amplitude and AUC by EA treatment (Figure 5C–J). These results suggest that EA exerts an inhibitory effect on the abnormal functional plasticity of spinal excitatory neurons under the postoperative pain state.

This assumption was further strengthened by the in vivo real‐time miniscope GRIN lens Ca^2+^ imaging at a single neuron level in the SDH (Figure 5K). Visualization of a single spinal excitatory neuron under a miniscope GRIN lens demonstrated that a number of GCaMP6s‐expressing spinal excitatory neurons exhibit obvious Ca^2+^ transients in response to pinch stimulation of the cutaneous receptive field in the control state (Figure 5L–O; Videos S9–S11, Supporting Information). Following PI injury, these stimulus‐evoked Ca^2+^ responses were greatly potentiated in both the peak amplitude and AUC (Figure 5L–O). Delivery of EA treatment profoundly reversed this functional potentiation associated with PI injury (Figure 5L–O). Overall, these results provide convincing evidence that EA treatment is able to relieve the spinal sensitization through LC top‐down noradrenergic projections under the postoperative pain state.

### 2.6 EA Stimulation Normalizes the Dysregulated α_2A_‐Adrenoceptor‐CaMKII Signaling Cascades in the SDH after PI Injury

**Figure 6:**
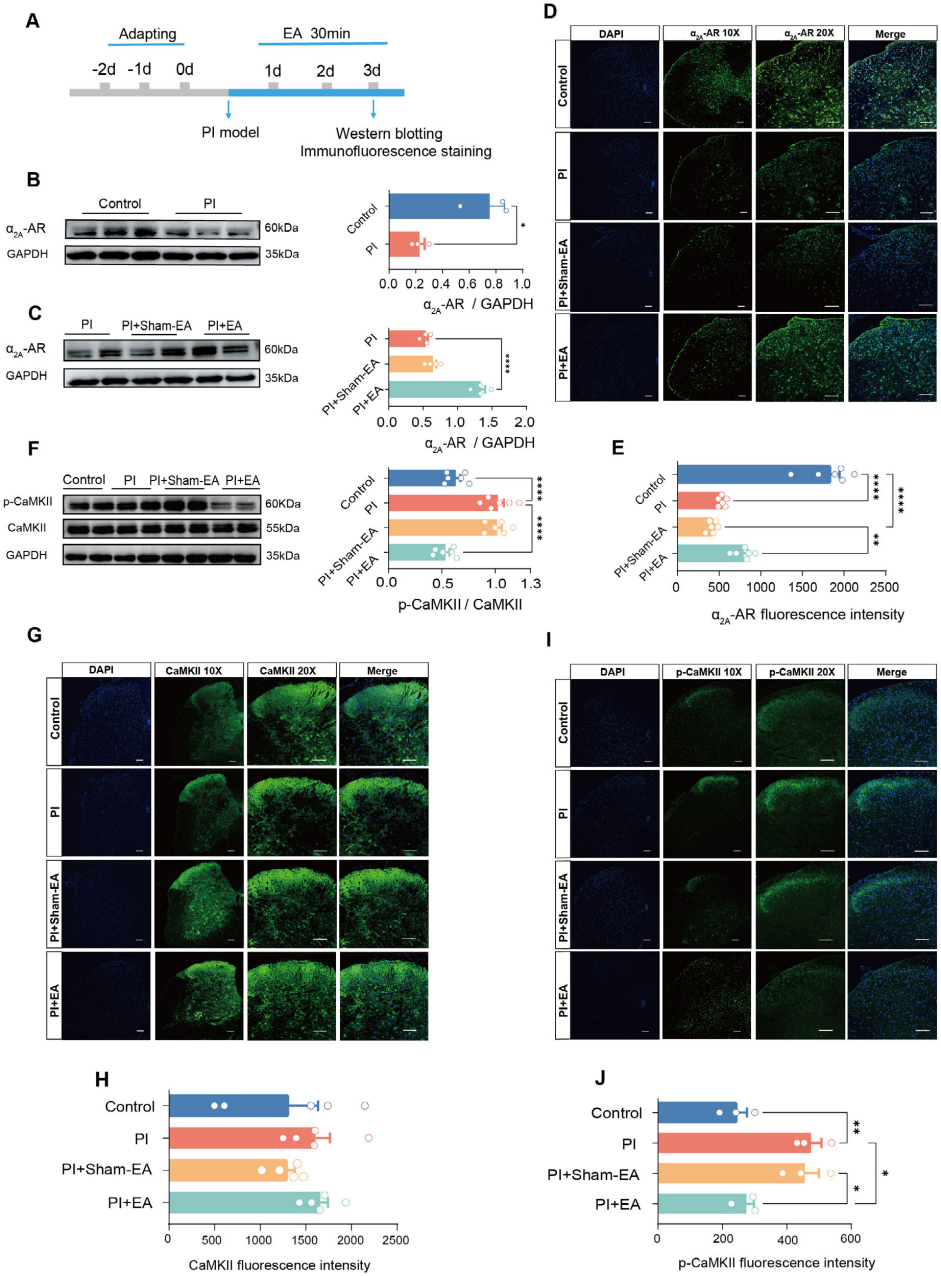
EA stimulation normalizes the dysregulated α_2A_‐adrenoceptor ‐CaMKII signaling cascades in the SDH after PI injury. A) Experimental schematic diagram for western blotting and immumofluorescence staining. B,C) Representative blots and quantitative summary (B,C,F) showing α_2A_ ‐ adrenoceptor expression (B, C) at the protein level in the SDH in control, PI‐injured, and PI‐injured mice receiving EA or sham‐EA treatment (per group, *n* = 3 samples of 6 mice). *p < 0.05 by two‐tailed unpaired *t*‐test (B), ****p < 0.0001 by one‐way ANOVA followed by Tukey's multiple comparisons test HSD (C). D,E) Representative immunofluorescence photographs (D) and quantitative summary (E) showing anti‐α_2A_‐adrenoceptor immunoreactivity in the SDH in control, PI‐injured, and PI‐injured mice receiving EA or sham‐EA treatment (per group, *n* = 3 mice, total 6 sections/group). Scale bars  =  50 µm. **p < 0.01, ****p < 0.0001 by one‐way ANOVA followed Tukey's multiple comparisons test HSD (E). F) Representative blots and quantitative summary showing CaMKII and p‐CaMKII expression at the protein level in the SDH in control, PI‐injured, and PI‐injured mice receiving EA or sham‐EA treatment (per group, *n* = 6 samples of 12 mice). ****p < 0.0001 by one‐way ANOVA followed Tukey's multiple comparisons test HSD (F). G–J) Representative immunofluorescence photographs (G,I) and quantitative summary (H,J) showing anti‐CaMKII (G,H) and p‐CaMKII (I, J) immunoreactivity in the SDH in control, PI‐injured, and PI‐injured mice receiving EA or sham‐EA treatment (per group, *n* = 5 mice, total 6 sections/group). Scale bars  =  50 µm. *p < 0.05, **p < 0.01 by one‐way ANOVA followed Tukey's multiple comparisons test HSD (J). Data are represented as mean ± S.E.M. See Table S2 (Supporting Information) for detailed statistical information.

Then, what could be the molecular mechanisms underlying EA‐induced analgesia via activation of LC noradrenergic‐spinal projections? NE release from LC exerts the modulatory role in various brain functions, including nociception, emotion, and cognition via interacting with its corresponding noradrenergic receptors.^[^ ^32^, ^33^, ^34^ ^]^ Amongst which, α_2A_‐adrenoceptor has been reported to be highly expressed in the SDH and has been linked with the antinociceptive effects of NE.^[^ ^32^, ^33^, ^34^, ^35^, ^36^ ^]^ We then asked whether EA protects against postoperative pain via modulation of spinal α_2A_‐adrenoceptor. To this end, we employed several approaches to examine the changes of spinal noradrenergic receptors and their downstream signaling cascades after PI injury and EA treatment (schematic diagram shown in **Figure** **6**A). As shown in Figure 6B, western blotting analysis revealed that the expression of α_2A_‐adrenoceptor in the SDH was significantly downregulated after PI injury as compared to the control group (Figure 6B). In comparison with sham‐EA treatment, EA stimulation greatly reversed the PI‐induced downregulation of α_2A_‐adrenoceptor in the SDH (Figure 6C), indicating the possible involvement of spinal α_2A_‐adrenoceptor in EA‐induced antinociception. This was further supported by the immunofluorescence staining. We observed that the immunoreactivity of α_2A_‐adrenoceptor is strong in the SDH of control mice, which was significantly reduced upon PI injury (Figure 6D,E). This PI‐induced reduction of α_2A_‐adrenoceptor immunoreactivity in the SDH was nicely normalized by EA stimulation (Figure 6D,E). To further confirm the action of spinal α_2A_‐adrenoceptor in the EA‐induced antinociception, in parallel, we observed the changes of other subtypes of stimulatory adrenoceptors, α_1A_‐adrenoceptor, α_1B_‐adrenoceptor, and β_2_‐adrenoceptor, which are assumed to be pronociceptive in the SDH.^[^ ^32^, ^37^, ^38^, ^39^, ^40^, ^41^ ^]^ In contrast, the expression of either α_1A_‐adrenoceptor, α_1B_‐adrenoceptor, or β_2_‐adrenoceptor was not altered in the SDH upon PI treatment (Figure S4A–C, Supporting Information). EA delivery did not cause the changes in them as well (Figure S4A–C, Supporting Information). This indicates that amongst adrenergic receptor subtypes, PI injury may preferentially lead to the downregulation of α_2A_‐adrenoceptor in the spinal dorsal horn, which is effectively normalized after EA treatment and results in antinociception.

Noradrenergic receptors have been shown to interact with calcium/calmodulin‐dependent protein kinase II (CaMKII) signaling in the mediation of various pathophysiological processes, including pain sensitization, cardiac contractility, etc.^[^ ^42^, ^43^ ^]^ Considering the crucial role of CaMKII in the spinal sensitization and functional plasticity involved in the pathological pain processing, we went on to examine whether CaMKII acts as a downstream target of α_2A_‐adrenoceptor in the EA‐induced analgesia. Western blotting analysis demonstrated that PI injury did not induce significant changes of CaMKII in the SDH, but did upregulate the phosphorylation of spinal CaMKII (p‐CaMKII) (Figure 6F). EA stimulation but not sham‐EA treatment markedly reversed this enhanced p‐CaMKII expression (Figure 6F). We further verified the above observations using immunofluorescence staining. As shown in Figure 6G,H, CaMKII immunoreactivity in the SDH was not different in control and PI‐injured mice receiving EA or sham‐EA treatment. In contrast, immunoreactivity of p‐CaMKII was markedly increased in the superficial dorsal horn after PI injury (Figure 6I,J). This enhanced p‐CaMKII immunoreactivity was significantly attenuated by EA stimulation but not by sham‐EA treatment (Figure 6I,J). In sum, these results collectively suggest that EA produces antinociception probably by acting to enhance the LC noradrenaline‐spinal α_2A_‐adrenoceptor system and further inhibit its downstream cascade CaMKII signaling.

### 2.7 Blockade of Spinal α_2A_‐Adrenoceptor with Yohimbine Significantly Reverses EA‐Induced Downregulation of p‐CaMKII, Depression of Spinal Neuronal Activity, as well as Antinociception

**Figure 7:**
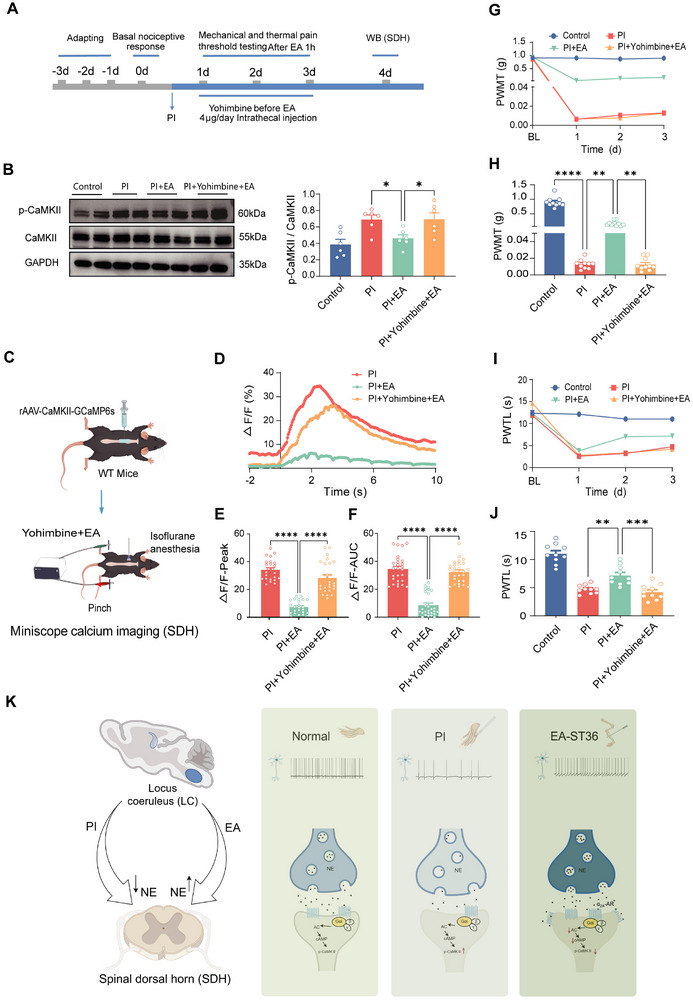
Blockade of spinal α_2A_‐adrenoceptor with yohimbine significantly reverses EA‐induced downregulation of p‐CaMKII, depression of spinal neuronal activity, as well as pain relief. A) Experimental schematic diagram for pharmacological intervention, behavioral testing, and western blotting assay. B) Representative blots and quantitative summary showing CaMKII and p‐CaMKII expression at the protein level in the SDH in control, PI‐injured, and PI‐injured mice receiving EA in the absence and presence of yohimbine (per group, *n* = 6 samples of 12 mice). *p < 0.05 by one‐way ANOVA followed Dunnett's multiple comparisons test HSD (B). C) Schematic diagram showing in vivo GRIN lens miniscope calcium imaging in the SDH. D) Typical examples of traces, quantitative summary of peak amplitude E) and area under curve (AUC) F) showing Ca^2+^ transients in spinal excitatory neurons induced by application of pinch stimulation to the cutaneous receptive field in PI‐injured and PI‐injured mice receiving EA treatment in the absence and presence of yohimbine. *n* = 24 cells from 3 mice per group. ****p < 0.0001 by the Kruskal–Wallis H test followed Dunn's multiple comparisons test HSD (E, F). G–J) Time course and magnitude of mechanical threshold and thermal latency (H, J) showing the effect of yohimbine on EA‐induced inhibition of mechanical allodynia (G, H) and thermal hyperalgesia (I, J) in postoperative pain (per group, *n* = 10 mice). **p < 0.01, ***p < 0.001, ****p < 0.0001 by the Kruskal–Wallis H test (H) and One‐way ANOVA(J). K) A schematic model proposing how EA produces analgesia in postoperative pain by activating LC noradrenergic‐spinal projections (please see the main text for details). Data are represented as mean ± S.E.M. See Table S2 (Supporting Information) for detailed statistical information.

In order to confirm that the above top‐down signaling cascades underlie EA‐induced antinociception in postoperative pain, pharmacological intervention was combined with in vivo real‐time spinal miniscope GRIN lens calcium imaging and behavioral surveys (**Figure** **7**A). As described above, EA stimulation significantly eliminated the PI‐induced increase of p‐CaMKII in the SDH (Figure 7B). This EA effect was reversed by intrathecal administration of yohimbine, an antagonist of α_2A_‐adrenoceptor (Figure 7B). CaMKII has been shown to be a key determinant for spinal neuronal excitability, synaptic plasticity, and pain sensitization.^[^ ^42^, ^44^, ^45^, ^46^, ^47^ ^]^ We further tested whether a blocker of α_2A_‐adrenoceptor is able to influence the role of EA on the enhanced spinal neuronal activity under postoperative pain state using miniscope GRIN lens spinal Ca^2+^ recording at single neuron level (Figure 7C,D). Photometric Ca^2+^ signals in single spinal excitatory neurons revealed that topical spinal administration of yohimbine substantially relieved the inhibitory effect of EA on mechanical pinch‐evoked Ca^2+^ responses in both the peak amplitude and AUC after PI injury (Figure 7E,F). This results in a behavioral manifestation that intrathecal delivery of yohimbine excluded the EA‐induced inhibition on the mechanical allodynia and thermal hyperalgesia associated with PI injury (Figure 7G–J). Taken together, these results suggest that EA‐evoked antinociception in postoperative pain is dependent on activation of LC noradrenaline‐spinal α_2A_‐adrenoceptor ‐CaMKII signaling cascades.

## 3 Discussion

The results of the present study led us to propose the model represented schematically in Figure 7K. Following PI, the activity of noradrenergic neurons in the LC gets downregulated, which brings about the impaired release of NE in the SDH. Delivery of EA treatment restores the dysregulated hypoexcitable state of LC noradrenergic neurons after PI injury and thus triggers enhanced NE release in the SDH. The resultant increased NE release in the SDH activates α_2A_‐adrenoceptor and inhibits CaMKII signaling, which in turn depresses spinal excitatory neuronal hyperexcitability and eventually relieves postoperative pain. Thus, this study primarily clarifies how EA produces analgesia via activation of LC noradrenergic‐spinal projections in postoperative pain.

### 3.1 PI Impairs LC Noradrenergic Neuronal Activity and NE Release

Although acupuncture has been proven to be a potent therapeutic strategy for pain treatment in clinical practice in multiple types of pain, including postoperative pain, for over 3000 years,^[^ ^3^, ^6^, ^7^, ^8^, ^9^, ^10^ ^]^ the mechanisms that lie behind it are not fully understood. Over the last several decades, although peripheral and spinal mechanisms have been gradually revealed,^[^ ^10^, ^48^ ^]^ very little is known regarding the brain mechanism responsible for the antinociceptive action of EA. To this end, we performed an unbiased whole‐brain screening for mapping the potential brain regions in response to EA treatment using a widely used neuronal activity marker, c‐Fos. Our observations showed that a number of brain regions display increased c‐Fos immunoreactivity after EA administration, including M1, M2, V1, amygdala, thalamus, hypothalamus, PAG, LC, and RVM. Amongst which, activation of LC brought to our attention based on two aspects, one is previous studies showing the involvement of neurochemicals NE in the action of EA,^[^ ^10^ ^]^ the other is considering that LC is the main source of NE in the CNS and is involved intensively in modulating pain and stress‐related disorders (e.g., major depressive disorder and anxiety) and in their comorbidity.^[^ ^33^ ^]^ However, several questions remain open. For example, how is LC exactly engaged in postoperative pain? How is LC modulated by EA? Is the LC the main source of NE in the antinociceptive action of EA?

Previous studies have shown that LC gets activated by acute harmful noxious stimuli, inflammation, or nerve damage, promoting feedback inhibition of pain, as characterized by increased c‐Fos expression induced by these irritants in the LC.^[^ ^49^, ^50^, ^51^ ^]^ Unexpectedly, our results showed that following PI, c‐Fos expression in TH‐positive neurons in the LC displays marked downregulation, suggesting the downregulated activity of LC noradrenergic neurons under the postoperative state. This was further supported by our electrophysiological recordings. Spontaneous firing is the main nature of LC noradrenergic neurons, which triggers the release of NE for the modulation of a variety of brain functions.^[^ ^33^, ^52^ ^]^ Consistent with the downregulated c‐Fos expression, we observed that both the incidence rate of neurons showing spontaneous firing and the frequency of spontaneous firing in LC noradrenergic neurons are profoundly inhibited in PI‐injured mice as compared to control mice. Furthermore, in a subgroup of LC noradrenergic neurons without spontaneous firing, I–O curve results showed the decreased firing frequency and heightened rheobase in PI‐injured LC noradrenergic neurons in response to depolarizing current injection as compared to control neurons. These lines of evidence provide us a strong hint that PI injury drives the LC noradrenergic neurons into a hypoexcitable state. This is in contrast with a previous study showing the enhanced excitability in LC neurons after intraplantar formalin injection into the rat hindpaw.^[^ ^53^ ^]^ The discrepancy between this prior study and our study might be due to the difference in animal pain models and species used. The resultant hypoexcitability of LC noradrenergic neurons after PI injury caused the impaired NE release, as revealed by the ELISA assay and in vivo real‐time NE release monitoring using GRAB_NE_ sensors in the SDH. Overall, this is one intriguing finding of this study that we depicted for the first time: the dysregulated functional plastic changes of LC noradrenergic neurons under the postoperative state. Given the crucial role of the LC‐noradrenergic system in descending pain control, this downregulated functional activity of LC noradrenergic neurons after PI injury may reflect the impaired tonic descending inhibition from LC, which in turn leads to pain sensitization.

### 3.2 EA Produces Antinociception in Postoperative Pain via Rectifying the Defect of LC Noradrenergic Neuronal Activity

Then, how would EA modulate the above abnormal functional plasticity of LC noradrenergic neurons associated with PI injury? Collective evidence from c‐Fos staining and electrophysiological recording revealed that the hypoactivity of LC noradrenergic neurons induced by PI injury is greatly restored after EA administration, as characterized by increased c‐Fos expression in TH‐positive neurons, enhanced spontaneous firing as well as exaggerated excitability in response to depolarizing current injection in LC noradrenergic neurons derived from PI‐injured mice receiving EA delivery. These results infer that LC noradrenergic neurons get activated by EA administration under the postoperative pain state. This is in agreement with some studies reporting LC activation by EA,^[^ ^16^, ^17^, ^18^, ^19^, ^20^ ^]^ but in contrast with other studies showing LC deactivation by EA.^[^ ^21^, ^22^, ^23^, ^24^ ^]^ In further support of the activation of LC by EA after surgical incision, we performed in vivo real‐time monitoring of LC noradrenergic neuronal activity during EA administration by using fiber photometry recording and found that EA stimulation induces persistent Ca^2+^ elevation in LC noradrenergic neurons. To the best of our knowledge, this is the first direct and real‐time evidence showing that LC noradrenergic neurons get activated by EA administration, which in turn aids in restoring the defect of LC noradrenergic neuronal plasticity after surgical incision.

LC is assumed to be intensively involved in the pain processing.^[^ ^33^ ^]^ Given the above observation of LC activation by EA, an interesting question that ensues is whether activation of LC noradrenergic neurons is required for EA‐induced antinociception in postoperative pain. Interestingly, we did observe that optogenetic inhibition of LC noradrenergic neurons profoundly alleviates the inhibitory action of EA on mechanical allodynia and thermal hyperalgesia associated with PI injury, whereas optogenetic activation of LC noradrenergic neurons mimics the antinociceptive effect induced by EA administration. Taken together, these results indicate that EA produces antinociception in postoperative pain via rectifying the defect of LC noradrenergic neuronal plasticity.

Nevertheless, despite the above new insights, a very interesting question remains regarding the way how EA stimulation at ST36 acupoint leads to the changes of LC noradrenergic neurons. Recently, an elaborate study demonstrated that PROKR2^Cre^‐marked sensory neurons, which innervate the deep hindlimb fascia, are crucial for driving ST36 stimulation‐induced antiinflammation via vagal–adrenal axis.^[^ ^54^ ^]^ Traditional anterograde tracing with PHA‐L and physiological evidence showed that lamina I neurons of the spinal or medullary dorsal horn send direct projections or indirect projections via nucleus paragigantocellularis to the LC in animals, including rat, cat, and monkey.^[^ ^55^, ^56^ ^]^ Given these lines of evidence, it provides us with a possibility that ST36 stimulation may activate PROKR2^Cre^‐marked sensory neurons, their central terminals make synapses with spinal dorsal horn neurons, which further project directly or indirectly via nucleus paragigantocellularis to the LC and eventually lead to the changes of LC adrenergic neurons. However, this possibility remains to be further investigated in future studies.

### 3.3 EA‐Induced Antinociception is Dependent on LC Noradrenergic‐Spinal Projections and Subsequent α_2A_‐Adrenoceptor–CaMKII Signaling Cascades

After getting to know how EA modulates the abnormal functional plasticity of LC noradrenergic neurons, we then turned to figure out by which mechanism EA regulates pain hypersensitivity via the LC noradrenergic system. LC is known to send intensive efferents to an array of brain regions and the spinal cord in the regulation of a variety of brain functions, including emotion, cognition, nociception, etc.^[^ ^33^ ^]^ Amongst which, SDH is the first relay station in transmitting nociceptive signals from the periphery to CNS. Meanwhile, nociceptive sensory inflow in the SDH undergoes descending modulation from supraspinal regions, including LC noradrenergic projections, which is important for the homeostasis of the pain state.^[^ ^26^, ^28^ ^]^ Another intriguing finding of this study is that we revealed that EA produces antinociception via regulation of top‐down LC noradrenergic‐spinal projections and subsequent α_2A_‐adrenoceptor–CaMKII signaling cascades. Anatomical evidence has shown that there exists extensive noradrenergic termination in the SDH.^[^ ^57^, ^58^, ^59^ ^]^ Activation of LC noradrenergic neurons triggers NE release. Given the aforementioned restoration of neuronal excitability of LC noradrenergic neurons by EA administration, we would expect that EA administration could restore the impaired NE release after PI injury in the SDH. This possibility was exactly supported by our observations using the ELISA assay and in vivo real‐time NE release monitoring via GRAB_NE_ sensor in the SDH. These two compensatory approaches collectively showed that EA stimulation greatly increases the NE release in the SDH in PI‐injured mice. The resultant increase of NE release after EA administration significantly eliminated the exaggerated excitability of spinal excitatory neurons induced by PI injury. This is characterized by the dramatic inhibition of mechanical and thermal stimuli‐evoked Ca^2+^ elevation after EA treatment in PI‐injured mice at populated and single neuron levels, respectively, by using spinal fiber photometry recording and miniscope GRIN lens Ca^2+^ imaging. This led us to infer that under the postoperative state, EA treatment can help to rectify LC noradrenergic neuronal defects, which in turn increases NE release and reinstates LC‐triggered descending inhibitory control of spinal nociceptive inflows, thus alleviating pain hypersensitivity.

Then, what could be the molecular basis for the above processes? Evidence has shown that the α_2A_‐adrenoceptor is predominantly implicated in the antinociceptive effect of NE in the spinal cord.^[^ ^60^, ^61^, ^62^ ^]^ For example, Patel et al. reported that activating the α_2A_‐adrenoceptor in the spinal cord is able to reduce the hyperexcitability of SDH neurons and mitigate neuropathic pain.^[^ ^62^ ^]^ This suppressive effect of NE on pain transmission was reported to come about by interacting with α_2A_‐adrenoceptor expressed on the central terminals of nociceptors (presynaptic inhibition) as well as on postsynaptic spinal pain‐relay neurons (postsynaptic inhibition).^[^ ^32^, ^63^, ^64^, ^65^ ^]^ Whether EA produces analgesia in postoperative pain via modulation of spinal α_2A_‐adrenoceptor has remained elusive. In the present study, we observed that PI injury induces downregulation of α_2A_‐adrenoceptor in the SDH, which is normalized by EA administration. In contrast, we observed no changes of other subtypes of stimulatory adrenoceptors after PI challenge and EA stimulation, i.e., α_1A_‐adrenoceptor, α_1B_‐adrenoceptor, and β_2_‐adrenoceptor, which are assumed to exert the pronociceptive effect.^[^ ^32^, ^37^, ^38^, ^39^, ^40^, ^41^ ^]^ These results indicate that EA administration may act to increase both the NE release and its interacting α_2A_‐adrenoceptor, through which to inhibit the spinal hyperexcitability and thereby reduce pain sensitization. Calcium/calmodulin‐dependent protein kinase II (CaMKII) is one of the most abundant proteins found at the postsynaptic density. It has been widely reported that autophosphorylated CaMKII and synaptic translocation initiate multiple signaling cascades that elucidate the induction and maintenance of long‐term potentiation (LTP).^[^ ^47^, ^66^, ^67^ ^]^ Furthermore, in the pain‐related SDH, noxious stimulation leads to sustained activation of CaMKII. Inhibition of CaMKII activity abolishes spinal cord LTP,^[^ ^46^ ^]^ and reduces pain hypersensitivity resulting from peripheral tissue and nerve damage.^[^ ^68^, ^69^, ^70^ ^]^ Adding to this, our western blot and immunofluorescence staining results revealed that phosphorylated CaMKII (p‐CaMKII) was significantly elevated in the superficial SDH after PI injury, although no change of total CaMKII expression was seen. This enhanced p‐CaMKII in PI‐injured mice was largely eliminated by EA treatment, suggesting the regulatory role of EA on the spinal α_2A_‐adrenoceptor–CaMKII signaling cascades. Overall, these results suggest that EA‐induced NE release from LC noradrenergic neurons may directly act on the α_2A_‐adrenoceptor in SDH neurons, initiating intracellular signaling cascades that inhibit CaMKII autophosphorylation, thereby reducing neuronal hyperexcitability and diminishing pain signal transmission. This assumption was further supported by the pharmacological intervention that blockade of spinal α_2A_‐adrenoceptor with yohimbine, an antagonist of α_2A_‐adrenoceptor, markedly excluded EA‐induced inhibition of p‐CaMKII, enhancement of spinal neuronal activity, as well as antinociception.

In summary, this study sheds new light on the functional capability of LC noradrenergic‐spinal projections in EA‐induced analgesia in postoperative pain. We provide a better understanding of how surgical incision leads to the maladaptive plasticity of LC noradrenergic‐spinal projections and how EA regulates pain hypersensitivity by rectifying the defect of LC noradrenergic‐spinal projections at the cellular and molecular levels.

## 4 Experimental Section

### Animals

Experiments were conducted using adult mice (C57BL/6J mice or Dopamine‐β‐hydroxylase (DBH) – Cre mice, 6–8 weeks old) of both sexes, with an identical number of females and males in each group to minimize the possible gender difference. Mice were housed up to 5 per cage and maintained on a 12 h light/dark cycle with ad libitum access to food and water. All animal procedures were reviewed and approved by the Institutional Animal Care and Use Committee of the Fourth Military Medical University (FMMU, IACUC‐ 20230115). All testing was done in a double‐blinded manner. Animals were randomly allocated to different experimental groups. Animals undergoing surgery were sutured with administration of about 200 U/g Penicillin sodium and mildly kept on a heating pad after operation to keep the body temperature until they were awake. The operated animals were housed in plastic boxes separately with food and water available ad libitum in the colony room. All efforts were made to minimize the sacrificing of animals used.

### Postoperative Pain Model

The postoperative pain induced in this study involved PI of both the skin and muscle, as described previously.^[^ ^71^ ^]^ Anesthesia was initially obtained with 3–4% isoflurane laced with oxygen, which was then maintained with 1–2% isoflurane laced with oxygen. A surgical incision was made in the left hind foot of a mouse, ≈5 mm long along the midline from the plantar heel to the center of the plantar foot. The most important step is to elevate the flexor digitorum brevis muscle by inserting one end of the curved forceps underneath the lateral edge of the flexor digitorum brevis muscle and pushing the forceps through to the medial side of the muscle. The wound was sutured by removing the curved forceps from underneath the muscle and elevating the edges of the skin surrounding the wound with forceps. Close the wound by putting two sutures in the skin (but not muscle) ≈2 mm apart using 4‐0 nylon sutures and a hemostat. For control, a sham surgery was performed by anesthetizing the mouse, sterilizing the hindpaw, and applying erythromycin ointment to the plantar hindpaw.

### EA Intervention

The mice were maintained with 1–2% isoflurane laced with oxygen. Acupuncture needles (0.16 × 7 mm diameter) were inserted at a depth of 4 mm into bilateral Zusanli (ST36, 5 mm lateral to the anterior tubercula of the tibia; BL60, between the lateral malleolus and the Achilles tendon of the hind limb). A sham EA group animal received needle insertion subcutaneously into ST36 without electrical stimulation. All other groups of mice received the same treatment of isoflurane anesthesia as the EA or sham EA group, and the whole observation was the same as the EA group. The needles were connected with a HANS acupuncture point nerve stimulator (HANS‐200A Ji sheng Co., Ltd., Nanjing, China). Intervention parameters: 2/100 Hz alternating frequency, 2 mA stimulating intensity, 30 min intervention/session.

### Behavioral Analyses

Mice were adapted to the environment for 3 days prior to the testing and performed 1 h after the EA Intervention. Mice were placed in a plastic chamber on an elevated wire grid and acclimated for at least 30 min to the testing environment prior to the experiment. All testing was conducted in a blinded manner.

### Mechanical Allodynia

Mechanical withdrawal threshold testing (von Frey test) was conducted using calibrated von Frey filaments ranging from 0.008–2.0 g (0.008, 0.02, 0.04, 0.07, 0.16, 0.40, 0.60, 1.0, 1.4, and 2.0 g) on an elevated mesh‐bottomed platform (Danmic Global, CA, USA). Beginning with 0.008 g, filaments were applied to the 2 mm proximal to the site of PI treatment with just enough force to bend the fiber and held for 1 s. A “positive response” to the von Frey mechanical stimuli was defined as an abrupt foot lift upon application of von Frey hairs. Each filament was applied 10 times, and the paw withdrawal response frequency was recorded. The force of a particular filament required to elicit a 50% frequency of paw withdrawal was expressed as the mechanical threshold.

### Thermal Allodynia

Hargreaves test was performed on mice to assess thermal hyperalgesia. As a precaution against tissue damage, the Hargreaves apparatus was set at 25% intensity, and 20 s was established as a cutoff time.

### Gait Assessment

The Gait of mice was monitored and then analyzed via CatWalk XT system (Noldus Information Technology, The Netherlands). All images were recorded by the camera mounted underneath the glass plate, defined as 20 × 10 cm, including five paw prints from each mouse, and processed by the system's software. The mice underwent three days of training, in which they were placed on one side of the track and allowed to run through the track without interruption, three times per day, before the formal tests. Data were expressed as different values of stride length, duration, stand, and swing speed before and after PI or EA treatment. For the detection of all parameters, the camera gain was set to 20 (dB) and the detection threshold to 0.1. For each animal and analyzed time point, 3 compliant runs with a run duration of 0.5–20 s and maximum allowed speed variation of 60%, were acquired per trial.

### ELISA Assay

Mouse serum and lumbar enlargement of SDH tissues were collected after EA or PI. The tissue samples were homogenized in a lysis buffer containing protease and phosphatase inhibitors. Protein concentrations were determined by BCA （bicinchoninic acid） Protein Assay (Pierce). NE (E‐EL‐0047) ELISA kit was purchased from Elabscience, Wuhan, China. Detection of NE was performed in accordance with the manufacturer's protocol. In brief, 50 µL standard product and samples were added into the appropriate wells separately. The 50 µL Biotinylated Detection antibody working solution was added into each well, incubating at 37 °C for 1 h. After washing, 100 µL HRP enzyme conjugate working solution was added for incubation at 37 °C for 30 min, followed by substrate solution (TMB) (90 µL) incubation at 37 °C for 15 min away from light. The reaction was terminated by adding 50 µL stop solution, and the O.D. absorbance at 450 nm was read immediately. The standard curve was drawn, and the concentration was calculated for each well.

### Western Blotting

Mouse SDH tissues were collected and homogenized in ice‐cold lysis buffer containing 50 mm Tris‐HCl, pH 7.4, 150 mm NaCl, 5 mM EDTA, 1% Triton X‐100, 0.5% sodium deoxycholate, 0.1% SDS, and standard protease inhibitors. Insoluble material was removed by centrifugation (15 000 rpm × 10 min), and the supernatant was collected. The total protein concentration was measured using the BCA Protein Assay Kit (23 225, ThermoFisher, USA), mixed with 5 × SDS‐PAGE loading buffer (CWBIO, Beijing, China), and then heated at 100 °C for 10 min. Protein samples were loaded and resolved by SDS‐PAGE, and immunoblotted with corresponding antibodies (See Table S1 for details). Primary antibodies, including rabbit anti‐α_2A_‐adrenoceptor 1:500, AP1‐048, ThermoFisher, USA), rabbit anti‐CaMKII (1:2500, ab52476, Abcam, Cambridge, UK), rabbit anti‐CaMKII alpha (phosphor T286) (1:2000, ab124880, Abcam, Cambridge, UK), and mouse anti‐GAPDH (1:50 000, Proteintech, China), were used at 4 °C and incubated overnight. The corresponding second antibody was incubated for 2 h at room temperature, and the protein was observed by chemiluminescence assay. The captured images were quantified using ImageJ software. The specific bands of each protein were normalized to their respective GAPDH.

### Immunofluorescence Labeling

Mice were anesthetized by intraperitoneal injection of 1% sodium pentobarbital and transcranial perfused with normal saline (20 ml) followed by 4% paraformaldehyde (PFA, 20 ml). The brain and spinal cord tissue was placed in 4% paraformaldehyde overnight and protected by freezing in 30% sucrose until it sank to the bottom of the container. Simply, sections were sliced on a cryostat with a thickness of 30 µm (brain 30 µm, spinal cord 15 µm) and immunostained with primary antibodies such as rabbit anti‐α_2A_‐adrenoceptor (1:100, AP1‐048, ThermoFisher, USA), rabbit anti‐CaMKII (1:250, ab52476, Abcam, UK), rabbit anti‐CaMKII alpha (phosphor T286) (1:500, ab5683, Abcam, UK), rabbit anti‐TH (1:200, A0028, Wuhan, China), guinea pig anti‐c‐Fos (1:2000, 226 308, Synaptic Systems, USA) at 4 °C 48 hours. Second antibodies, including Alexa Fluor 488 (donkey anti‐rabbit IgG, 1:800, Invitrogen, Carlsbad, USA), and Alexa Fluor 594 (donkey anti‐Guinea pig IgG, 1:800, Jackson ImmunoResearch, USA), were incubated for 3–4 h at room temperature. The nuclei were stained by DAPI (1:1000). All images were captured using an Olympus confocal microscope (Olympus FV3000, Japan). Captured images with a 20× magnification under this confocal microscope were processed using ImageJ. Quantification of cell counts was performed manually. Signal intensity was quantified as mean gray value using ImageJ software in at least seven sections from three to six animals. All counting experiments were conducted blinded to the experimental group.

### Stereotaxic Surgery

Stereotaxic surgery was performed as described previously.^[^ ^72^ ^]^ In brief, mice were maintained under anesthesia with 1%–2% isoflurane, and their heads were fixed in a stereotactic frame (RWD Life Science Inc., Shenzhen, China). Their eyes were applied to erythromycin to prevent corneal drying. All skull measurements were made relative to Bregma, and the virus was injected into the LC and SDH at a rate of 40 nL min^−1^ using a 10‐µL micro syringe (Gaoge, Shanghai, China) with a microelectrode to deliver the virus using a micro syringe pump. After viral injection, the microelectrode was kept in place for 10 min to allow diffusion of the virus. The stereotaxic coordinates for LC injection were anterior posterior (AP) 5.40, medial lateral (ML) 0.80, and dorsal ventral (DV) 3.80 mm. For calcium imaging of LC in DBH‐Cre mice, 200 nL of rAAV9‐EF1α‐DIO‐GCaMp6s was injected into the left LC, and the optical fiber (400 µm optical density, 0.37 numerical aperture) was implanted in the injected LC (AP 5.4 mm, ML 0.80 mm, DV 3.7 mm). For optogenetics in LC of DBH‐Cre mice, 200 nL of rAAV9‐EF1α‐DIO‐hChR2(H134R)‐mCherry or rAAV9‐EF1α‐DIO‐eNpHR3.0‐mCherry or rAAV9‐EF1α‐DIO‐mCherry was injected into the bilateral LC (AP 5.4 mm, ML 0.80 mm, DV 3.7 mm), and optical fiber was implanted in the injected LC with an angle of ±10° (AP 5.4 mm, ML 1.50 mm, DV 3.7 mm). Data were excluded from experiments when the viral injections were inaccurate.

For calcium imaging or NE release monitoring in the SDH, 500 nL of rAAV9‐CaMKII‐GCaMP6s or rAAV‐hSyn‐NE2h were slowly injected into the L3–L4 SDH segment. Twenty‐one days after stable virus expression, the GRIN lens miniscope electronic focus imaging system was used to locate the receptive field corresponding to the left plantar in the SDH. Titanium alloy needles and titanium wires were fixed into a frame structure. The optical fiber was connected to the multi‐channel fiber photometry device to find and fix the position with a good signal. The skin was sutured intermittently. Data were excluded from experiments when the viral injections were inaccurate. Details of virus information have been provided in Table S1.

### Fiber Photometry Recording and Miniscope GRIN Lens Calcium Imaging

Fiber photometry was used to record calcium‐dependent activity dynamics with the commercialized fiber photometry system (Inper Bioscience, Hangzhou, China) as described previously.^[^ ^73^ ^]^ An optical fiber was implanted into the LC and the surface of the SDH of mice. To excite GCaMP6s, a 473‐nm LED (Cree XP‐E LED, Shenzhen, China) was reflected off a dichroic mirror (MD498, Thorlabs, Newtown, NJ, USA) that was focused by a 20× objective lens (0.4 NA; Olympus, Ishikawa, Japan) and coupled to an optical commutator (Doris Lenses). An optical fiber (200 µm OD, 0.37 NA) guided the light between the commutator and the implanted optical fiber. The laser power at the tip of the optical fiber was adjusted to 0.01–0.02 mW to decrease laser bleaching. Fluorescence was bandpass‐filtered (MF525‐39, Thorlabs), and an amplifier was used to convert the CMOS (DCC3240M, Thorlabs) current output to signals, which were further filtered through a low‐pass filter (40 Hz cut off, ThinkerTech). The analog voltage signals were digitalized at 60 Hz and recorded by the multi‐channel fiber photometry recording system (ThinkerTech). Calcium signals analysis was performed using the Inper multi‐channel fiber optic recording system analysis software. We subtracted the scaled 410‐nm reference trace from the 470‐nm signal to obtain the motion‐corrected 470‐nm signal. We calculated the normalized change in motion‐corrected 470‐nm signal (△F/F) by subtracting the median signal from the signal at each time point and dividing that value by the median signal.

During fiber photometry recording in the LC (schematic diagram shown in Figure 2J), EA stimulation at ST36 acupoint was applied simultaneously for 30 min under anesthesia with 1%–2% isoflurane. A dual‐color multichannel fiber photometry system was used for consecutive recording for 30 min, and the EA ST36 lasted for 25 min.

For fiber photometry recording in the SDH in freely moving mice (schematic diagram shown in Figure 5B), mice were placed in a plastic chamber on an elevated wire grid and acclimatized for at least 30 min to the testing environment prior to the commencement of the experiment. Calcium transients in SDH neurons were induced by noxious mechanical and thermal stimulation, as applied by clamping a layer of skin with a small serrated clip and radiant heat resources from the Hargreaves apparatus, respectively. To avoid tissue damage, the Hargreaves apparatus was set at 25% in the light intensity and 20 s as a cutoff time.

For miniature microscope (miniscope) calcium imaging in the SDH (schematic diagram shown in Figure 5K), all calcium data were collected using a miniscope from Inscopix (nVista). The miniscope imaging method in the SDH is the same as fiber photometry recording described above, except that the vertebral body and optical fiber are no longer fixed. The miniscope was focused properly, and the field of view for imaging was established by using the nVista acquisition software. All tests were conducted in a blinded manner. Miniscope calcium signals in the SDH were induced by application of noxious mechanical and radiant heat stimulation to the cutaneous receptive field. Calcium data analysis was performed using the nVista acquisition software.

### NE Release Monitoring

In vivo real‐time NE release was monitored using a miniscope from Inscopix (nVista) (schematic diagram shown in Figure 4D) as shown above. In brief, the miniscope was focused properly, and the field of view was established by using the nVista acquisition software. All tests were conducted in a blinded manner. NE fluorescence signals were recorded in response to a variety of interventions. The forms of intervention used for quantification include: 1) EA treatment at ST36 acupoint with 2/100 Hz alternating frequency, 2 mA stimulating intensity, and 5 min duration; 2) sham‐EA treatment with only insertion of the acupuncture needle into ST36 without current stimulation; 3) optical activation of LC noradrenergic neurons with 488 nm laser; 4) optical inhibition of LC noradrenergic neurons with 589 nm laser and EA treatment. NE fluorescence analysis was performed using the nVista acquisition software.

### Optogenetic Manipulation

For optogenetic manipulation targeting LC noradrenergic neurons, an optic fibre canula (diameter of 200 µm, Newdoon) was implanted bilaterally into the brain area above 100 µm of the injection site. Dental cement was applied to make sure the implant was secured to the skull of the animal. Mice were connected with the optic cables, which are relayed by a rotatory joint, then connected to a 465 nm laser source or a 589 nm laser source (Inper, Hangzhou, China). The 5–8‐min pulse of blue light (465 nm, 10 mW, 20 Hz for noradrenergic neurons) or yellow light (589 nm, 10 mW, constant) was delivered and controlled using the Intelligent Optogenetics System (Inper, Hangzhou, China). The same stimulus protocol was applied for the control mice.

### Whole‐Cell Patch‐Clamp Recording in Brain Slices

A whole‐cell patch‐clamp recording was performed as described previously.^[^ ^74^ ^]^ Briefly, mice (4–6 weeks old) were anesthetized with 1% pentobarbital sodium and transcardially perfused with ice‐cold oxygenated (95% O_2_, 5% CO_2_) incubation solution (in mM: NaCl, 95; KCl, 1.8; KH_2_PO_4_, 1.2; CaCl_2_, 0.5; MgSO_4_, 7; NaHCO_3_, 26; glucose, 15; sucrose, 50; pH 7.4). The brain was removed and mounted on a vibratome (Leica1200s, Wetzlar, Germany), and coronal slices (300 µm thick) containing the LC (bregma −5.34 to −5.52 mm, determined by the shapes of lateral ventricles and corpus callosum) were prepared using the vibratome and stored in an incubation solution at room temperature. A slice was then transferred into a recording chamber and superfused with oxygenated recording solution at 1.5 mL min^−1^ at room temperature. The recording solution was identical to the incubation solution except for (in mM): NaCl 127, CaCl_2_ 2.4, MgSO_4_ 1.3, and sucrose 0. Standard whole‐cell patch clamp recordings were performed with glass pipettes having a resistance of 3 to 5 MΩ in LC noradrenergic neurons, which were identified by mCherry‐positive neurons via prior infusion of AAV2/9‐DIO‐mCherry into the LC of DBH‐Cre mice. Neurons were visualized with “Dodt” infrared optics using an X40 water‐immersion objective on an Olympus BX51 microscope equipped with a CCD camera. The pipette solution consisted of (in mM): K‐gluconate, 135; KCl, 5; CaCl_2_, 0.5; MgCl_2_, 2; EGTA, 5; HEPES, 5; and Mg‐ATP, 5, pH 7.4 with KOH, measured osmolarity 300 mOsm. The electrophysiological properties of the recorded neurons were acquired with an Axon700B amplifier (Molecular Devices Corporation, San Jose, CA) and pCLAMP10.0 software. Signals were low‐pass filtered at 5 kHz, sampled at 10 kHz, and analyzed offline.

For membrane properties analysis, depolarizing current steps (500 ms in duration and 50 pA increments) were injected to measure the AP parameters under current‐clamp mode. The AP threshold was determined by differentiating the AP waveform and setting a rising rate of 10 mV ms^−1^ as the AP inflection point. The AP amplitude was measured from the equipotential point of the threshold to the spike peak. The AP duration was measured at the half‐width of the spike.

### Pharmacological Intervention

Intrathecal administration was performed as described previously.^[^ ^75^ ^]^ In brief, mice were anaesthetized with 1% pentobarbital sodium. A midline incision was made along L2 to L4 vertebral plate, and the muscle attached to the spinous process was removed. With the tip of the sharp scissors, a 1‐mm hole on the left vertebra was made until dura and clean CSF were exposed. An intrathecal catheter (polyethylene‐10 tubing) was inserted from L3 and passed rostrally into the subarachnoid space until it reached L1/T13. After a flush with 10 µL saline, the exterior end of the catheter was sealed by heat. Penicillin antibiotics were used to prevent infection at the end of intrathecal catheterization. The mice were allowed to recover for 3 days. Any mouse showing motor deficits would be excluded. Yohimbine (4 µg/day) was intrathecally applied before EA treatment for the consecutive 3 days. For calcium imaging in SDH by miniscope electronic focus imaging system, Yohimbine was administered to the surface of spinal cord for incubation (4 µg/ mouse) for 10 min before recording.

### Data Analysis and Statistics

Data are analyzed in SPSS 27.0 and GraphPad Prism version 9.0. The normality test was performed by the Shapiro–Wilk test. The homogeneity of variance test was performed by Brown–Forsythe test. Data that met these two conditions were analyzed using a two‐tailed unpaired or paired *t*‐test, one‐way analysis of variance (ANOVA), or repeated‐measures ANOVA followed by Tukey's multiple comparisons test. Data sets that were not normally distributed were analyzed with a nonparametric test. Data are reported as mean ± SEM. A *p‐*value less than 0.05 was considered statistically significant. See Table S2 for detailed statistical information.

## Conflict of Interest

The authors declare no conflict of interest.

## Author Contributions

W.C, R.Z., and H.L. contributed equally to this work. W.G.C., R.Z., and T.H.L. conducted western blotting. W.G.C. and R.G.X. performed brain slice patch clamp recording. Y.C.L., H.T.L., W.G.C., and R.Z. performed animal preparation. W.G.C., R.Z., T.H.L., H.D., and Y.C.L. conducted immunofluorescence staining. W.G.C., R.Z., and X.X.Z. performed behavioral testing. W.G.C., R.Z., and F.W. conducted in vivo fiber photometry recording. W.G.C., R.Z., and Z.Z.L. performed NE imaging analysis and ELISA assay. W.G.C. and R.Z. conducted stereotaxic surgery. W.G.C., H.H.M., and H.D. analyzed the data. C.L. provided critical input on study design and interpretation. H.Y. provided comments on data interpretation. C.L. designed studies, C.L. and W.G.C. wrote the draft manuscript. C.L., R.G.X., and S.X.W. supervised the experiments and revised the manuscript. All the authors read and approved the final manuscript.

## Supporting information



Supporting Information

Supplemental Video 1

Supplemental Video 2

Supplemental Video 3

Supplemental Video 4

Supplemental Video 5

Supplemental Video 6

Supplemental Video 7

Supplemental Video 8

Supplemental Video 9

Supplemental Video 10

Supplemental Video 11

## Data Availability

The data that support the findings of this study are available from the corresponding author upon reasonable request.
