# Supplementary material for: Locus Coeruleus Noradrenergic‐Spinal Projections Contribute to Electroacupuncture‐Mediated Antinociception in Postoperative Pain in Mice
Source: Adv Sci (Weinh). 2025 May 19;12(25):e01182. doi: 10.1002/advs.202501182 (PMC12224967; doi:10.1002/advs.202501182)
Supplement: Supplementary file 1 — Supporting Information [file ADVS-12-e01182-s009.pdf]

ADVANCED SCIENCE
Open Access

Supporting Information

for *Adv. Sci.*, DOI 10.1002/advs.202501182

Locus Coeruleus Noradrenergic-Spinal Projections Contribute to Electroacupuncture-Mediated Antinociception in Postoperative Pain in Mice

Wen-Guang Chu, Ru Zhang, Hai-Tao Li, Ying-Chun Li, Hui Ding, Zhen-Zhen Li, Wen-Juan Han, Fei Wang, Xing-Xing Zheng, Hong-Hui Mao, Hua Yuan, Sheng-Xi Wu, Rou-Gang Xie* and Ceng Luo**

Supporting Information

Locus coeruleus noradrenergic-spinal projections contribute to electroacupuncture-mediated antinociception in postoperative pain in mice

Wen-Guang Chu[1#], Ru Zhang[1,2,3#], Hai-Tao Li[1,4#], Ying-Chun Li[1,5], Hui Ding[1], Zhen-Zhen Li[1], Wen-Juan Han[1], Fei Wang[1], Xing-Xing Zheng[1,5], Hong-Hui Mao[1], Hua-Yuan[6], Sheng-Xi Wu[1*], Rou-Gang Xie[1*], Ceng Luo[1,7*]

Fig S1

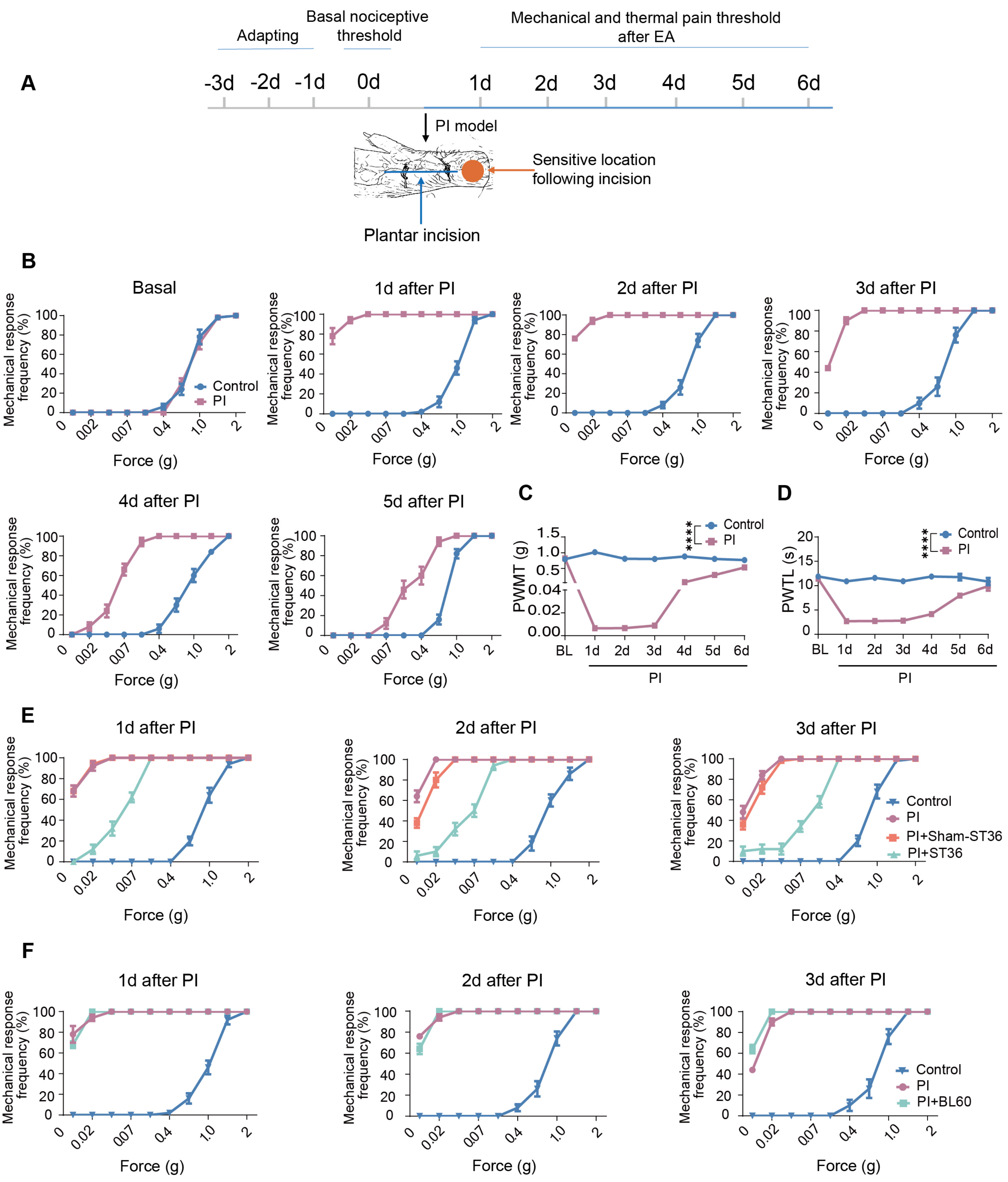
A
Adapting
Basal nociceptive threshold
Mechanical and thermal pain threshold after EA
-3d -2d -1d 0d 1d 2d 3d 4d 5d 6d
PI model
Sensitive location following incision
Plantar incision
B
Basal
1d after PI
2d after PI
3d after PI
4d after PI
5d after PI
Mechanical response frequency (%)
Force (g)
Control
PI
C
PWMT (g)
D
PWTL (s)
****
BL 1d 2d 3d 4d 5d 6d
PI
E
Control
PI
PI+Sham-ST36
PI+ST36
F
PI+BL60

Supplementary figure legends

Figure S1. (A) Experimental schematic diagram showing the establishment of PI model and behavioral testing. (B) Stimulus-response curve, mechanical threshold (C) and thermal latency (D) in response to von Frey hairs and radiant heat stimulation at basal and different time points after PI surgery (per group, n=10 mice). ****P < 0.0001 by Friedman's M test (C, D). (E) Stimulus-response curve in response to von Frey hairs showing the effect of EA treatment at ST36 acupoint in PI-induced mechanical allodynia (per group, n=10 mice). (F) Stimulus-response curve in response to von Frey hairs showing the effect of EA treatment at BL60 acupoint in PI-induced mechanical allodynia (per group, n=10 mice). Data are represented as mean ± S.E.M. See Supplemental Table 2 for detailed statistical information.

Fig S2

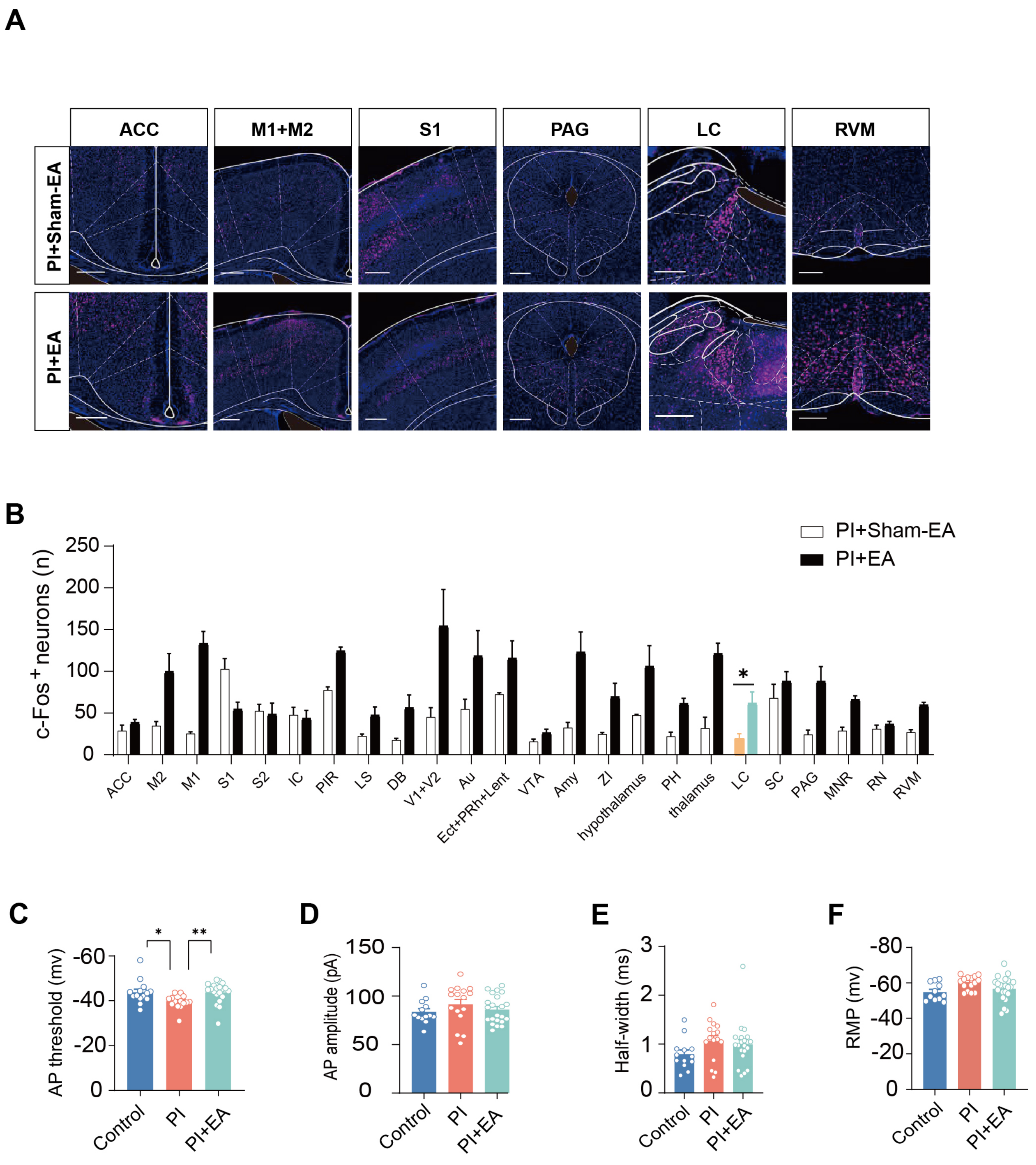
A
ACC
M1+M2
S1
PAG
LC
RVM
PI+Sham-EA
PI+EA
B
c-Fos+neurons (n)
250
200
150
100
50
0
PI+Sham-EA
PI+EA
*
ACC
M2
M1
S1
S2
IC
PIR
LS
DB
V1+V2
Au
Ect+PRh+Lent
VTA
Amy
ZI
hypothalamus
PH
thalamus
LC
SC
PAG
MNR
RN
RVM
C
AP threshold (mv)
-60
-40
-20
0
*
**
Control
PI
PI+EA
D
AP amplitude (pA)
150
100
50
0
Control
PI
PI+EA
E
Half-width (ms)
3
2
1
0
Control
PI
PI+EA
F
RMP (mv)
-80
-60
-40
-20
0
Control
PI
PI+EA

Figure S2. Representative immunofluorescence images (A) and quantitative summary (B) depicting the c-Fos expression in the ACC, M1+M2, S1, PAG, LC, RVM in mice following EA stimulation at ST36 acupoint and sham-EA treatment (per group, n=3 mice). Scale bars: 300 μm. *$P < 0.05$ by two-tailed unpaired t-test for LC. (C-F) Analysis of the other AP parameters including AP threshold (C), AP amplitude (D), AP half-width (E), and resting membrane potential (RMP) (F) in control, PI-injured and PI-injured mice receiving EA stimulation. *$P < 0.05$, **$P < 0.001$ by Kruskal-Wallis H test followed Dunn's multiple comparisons test HSD (C). Data are presented as mean ± SEM. See Supplemental Table 2 for detailed statistical information.

Fig S3

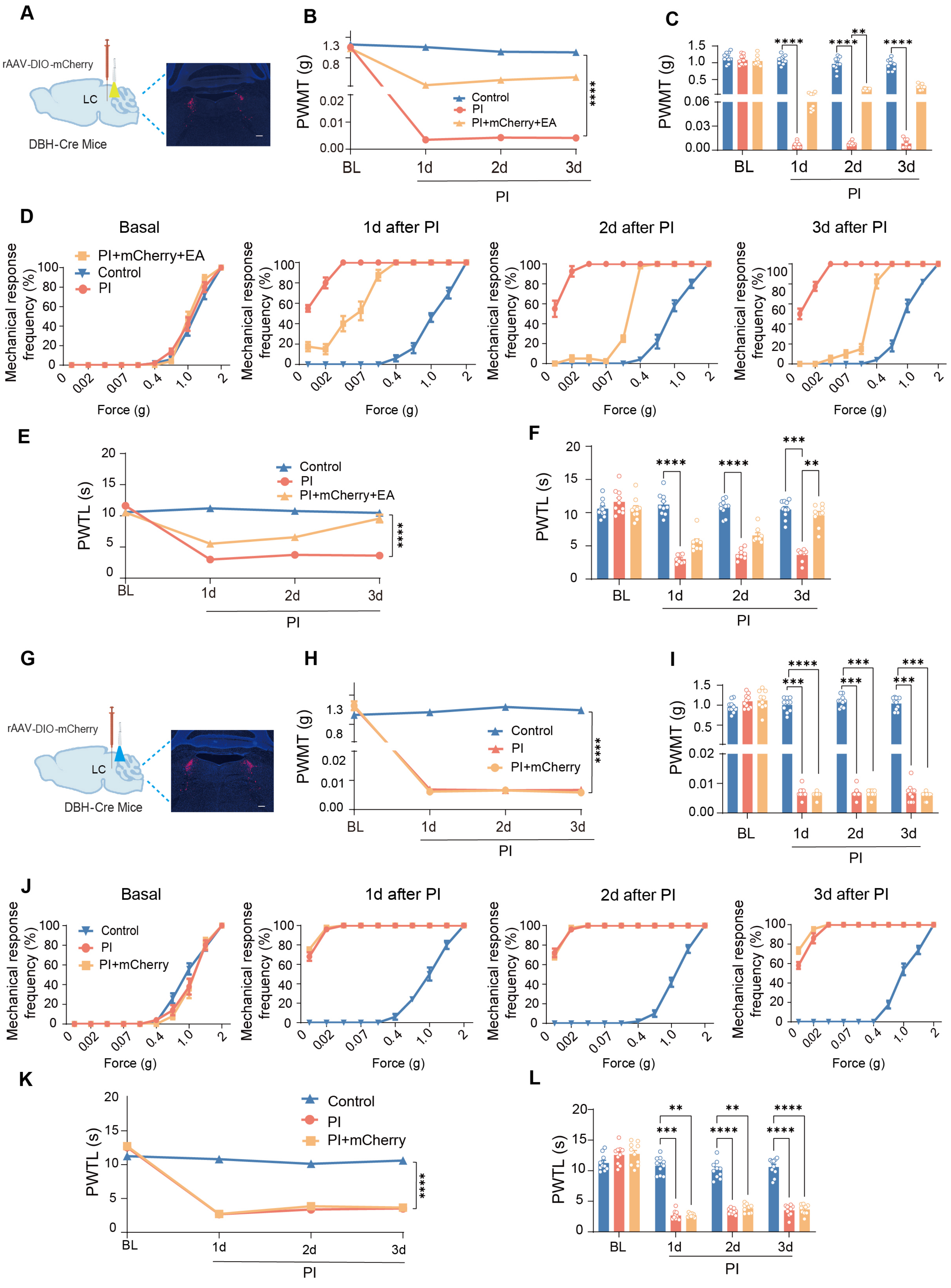
A
rAAV-DIO-mCherry
LC
DBH-Cre Mice
B
PWMT (g)
Control
PI
PI+mCherry+EA
****
BL
1d
2d
3d
PI
C
PWMT (g)
****
**
BL
1d
2d
3d
PI
D
Basal
1d after PI
2d after PI
3d after PI
PI+mCherry+EA
Control
PI
Mechanical response frequency (%)
Force (g)
E
PWTL (s)
Control
PI
PI+mCherry+EA
****
BL
1d
2d
3d
PI
F
PWTL (s)
****
***
**
BL
1d
2d
3d
PI
G
rAAV-DIO-mCherry
LC
DBH-Cre Mice
H
PWMT (g)
Control
PI
PI+mCherry
****
BL
1d
2d
3d
PI
I
PWMT (g)
****
***
BL
1d
2d
3d
PI
J
Basal
1d after PI
2d after PI
3d after PI
Control
PI
PI+mCherry
Mechanical response frequency (%)
Force (g)
K
PWTL (s)
Control
PI
PI+mCherry
****
BL
1d
2d
3d
PI
L
PWTL (s)
**
***
****
BL
1d
2d
3d
PI

Figure S3. Control experiments for optogenetic manipulation of LC noradrenergic neurons on EA-induced modulation of postoperative pain. (A) Schematic showing the control virus injection in the LC and fiber implantation. Scale bar: 500 μm. (B-F) Behavioral analysis showing light inhibition of control virus-injected LC in the effect of EA treatment in PI-induced mechanical allodynia and thermal hyperalgesia (per group, n=8-10 mice). $^{**}P < 0.01$, $^{****}P < 0.0001$ by Friedman's M test followed Dunn's multiple comparisons test HSD (B, C, E, F). (G) Schematic showing the control virus injection in LC and fiber implantation. Scale bar: 500 μm. (H-L) Behavioral analysis showing the role of light activation of control virus-injected LC in the PI-induced mechanical allodynia and thermal hyperalgesia (per group, n=8-10 mice). (per group, n=8-10 mice). $^{**}P < 0.01$, $^{***}P < 0.001$, $^{****}P < 0.0001$ by Friedman's M test followed Dunn's multiple comparisons test HSD (H, I, K, L). Data are represented as mean ± S.E.M. See Supplemental Table 2 for detailed statistical information.

Fig S4

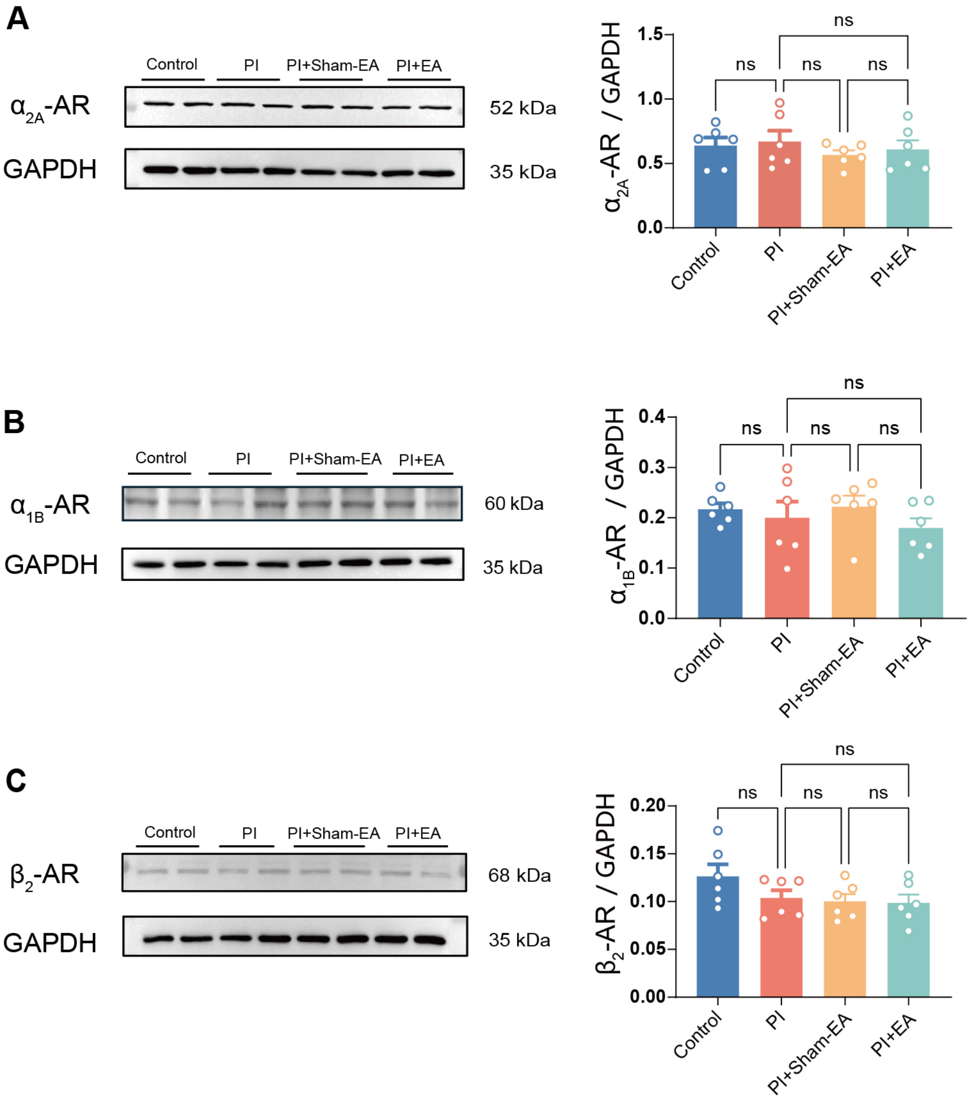
A
Control
PI
PI+Sham-EA
PI+EA
α2A-AR
GAPDH
52 kDa
35 kDa
α2A-AR / GAPDH
1.5
1.0
0.5
0.0
ns
ns
ns
ns
B
α1B-AR
60 kDa
α1B-AR / GAPDH
0.4
0.3
0.2
0.1
C
β2-AR
68 kDa
β2-AR / GAPDH
0.20
0.15
0.10
0.05
0.00

Figure S4. The changes of α_{1A}-adrenoceptor, α_{1B}-adrenoceptor or β_2-adrenoceptor expression after PI injury and EA treatment. Representative blots and quantitative summary showing α_{1A}-adrenoceptor (A), α_{1B}-adrenoceptor (B) or β_2-adrenoceptor (C) adrenoceptors expression at protein level in the spinal dorsal horn in control, PI-injured and PI-injured mice receiving EA or sham-EA treatment (per group, n=6 samples of 12 mice). A and C, ns: non significance by one-way ANOVA followed Tukey's multiple comparisons test HSD. B ns: non significance by Kruskal-Wallis test followed Dunn's multiple comparisons test.

Supplementary Table 1:Key resources of reagents and equipments

KEY RESOURCES TABLE

REAGENTS	SOURCE	IDENTIFIER	Dilution
Rabbit anti-TH	ABclonal, Wuhan, China	A0028	1:200 (IF)
Guinea pig anti-c-Fos	Synaptic System, Germany	226308	1:2000 (IF)
Rabbit anti-α_{2A}- AR	Invitrogen, Carlsbad, CA, USA	PA1-048	1:500 (WB)<br>1:100 (IF)
Rabbit anti-α_{1A}- AR	Abcam, Cambridgeshire, UK	ab137123	1:1000 (WB)
Rabbit anti-α_{1B} -AR	Affinity, China	DF8798	1:1000 (WB)
Rabbit anti-β_2 -AR	Abcam, Cambridgeshire, UK	ab182136	1:3000 (WB)
Rabbit anti-CaMKII	Abcam, Cambridgeshire, UK	ab52476	1:2500 (WB)<br>1:250 (IF)
Rabbit anti-p-CaMKII	Abcam, Cambridgeshire, UK	ab124880	1:2000 (WB)
Rabbit anti-p-CaMKII	Abcam, Cambridgeshire, UK	ab5683	1:500 (IF)
Mouse anti-GAPDH	Proteintech, Wuhan, China	66004-1-Ig	1:5000 (WB)
Anti-rabbit IgG,HRP-linked Antibody	Cell Signaling Technology, Boston, MA, USA	7074	1:4000 (WB)
Anti-mouse IgG,HRP-linked Antibody	Cell Signaling Technology, Boston, MA, USA	7076	1:4000 (WB)
Donkey anti-Rabbit IgG (H+L) Cross-Adsorbed Secondary Antibody, Alexa Fluor 488	Invitrogen, Carlsbad, CA, USA	A-1207	1:800 (IF)

Donkey anti-Guinea pig IgG (H+L) Cross-Adsorbed Secondary Antibody, Alexa Fluor 594	Jackson ImmunoResearch, USA	160797	1:800 (IF)
NE ELISA kit	Elabscience, Wuhan, China	E-EL-0047	
Chemicals, Peptides and Recombinant Proteins			
Yohimbine Hydrochloride	MedChemExpress, China	HY-N0127	
Formalin	Sigma-Aldrich, St. Louis, MO, USA	HT5012	
Isoflurane	RWD Life Science Inc., Shenzhen, China	N/A	
Virus Strains			
rAAV9-EF1α-DIO-GCaMp6s	Brain Case, Wuhan, China	BC-0086	
rAAV9-EF1α-DIO-eNpHR3.0-mCherry	Brain Case, Wuhan, China	BC-0126	
rAAV9-EF1α-DIO-mCherry	Brain Case, Wuhan, China	BC-0016	
rAAV9- CaMKIIα-GCaMP6s	Brain Case, Wuhan, China	BC-0081	
rAAV9-EF1α-DIO-hChR2(H134R)-mCherry	Brain Case, Wuhan, China	BC-0108	
rAAV9-hSyn-NE2h	Brain Case, Wuhan, China	BC-0268	
Experimental models: Organisms/Stains			
Mouse: DBH-cre	Tongji University, Dr. Ling Zhang	N/A	

Software		
Clampex 9.2	Axon Instrument	N/A
Clampfit 10.6	Axon Instrument	N/A
Olympus Fluoview version 3.1	Olympus	N/A
nVista acquisition software	Inscopix, Inc., USA	N/A
Inper Studio data acquisition	Inper Bioscience, Hangzhou, China	N/A
Other		
microsyringe pump	Gaoge, Shanghai, China	N/A
FV3000 laser-scanning confocal microscope	Olympus	N/A
dual-color multichannel fiber photometry system	Inper Hangzhou Bioscience Inc., Hangzhou, China	N/A
miniscope nVista	miniscope, Incopix Inc., USA	N/A
Model 400 heated base<br>Series 8 model 390G	IITC Life Science	Model 400
stereotactic frame	RWD Life Science Inc., Shenzhen, China	N/A
Anesthesia machine	RWD Life Science Inc., Shenzhen, China	N/A
von Frey Hairs	Bioseb	N/A
MultiClamp 700B	Axon Instrument	N/A

Supplementary Table 2: Statistical Methods

Data	Response variable	Groups	n define as	Animal used	Normality Test (Shapiro-Wilk)	Homogeneity of variance test (Brown-Forsythe test)	Hypothesis test	Test value	P value	Summary
**Fig1A**	PWMT(g)	Control-Basal (n=10), PI-Basal (n=10), PI+ST36-Basal (n=10), PI+Sham-ST36 Basal (n=10). Control-1d (n=10), Pl-1d (n=10), PI+ST36-1d (n=10), PI+Sham-ST36-1d (n=10). Control-2d (n=10), Pl-2d (n=10), PI+ST36-2d (n=10), PI+Sham-ST36-2d (n=10). Control-3d (n=10), Pl-3d (n=10), PI+ST36-3d (n=10), PI+Sham-ST36-3d (n=10). Control-4d (n=10), Pl-4d (n=10), PI+ST36-4d (n=10), PI+Sham-ST36-4d (n=10). Control-5d (n=10), Pl-5d (n=10), PI+ST36-5d (n=10), PI+Sham-ST36-5d (n=10). Control-6d (n=10), Pl-6d (n=10), PI+ST36-6d (n=10), PI+Sham-ST36-6d (n=10).	mouse	40 mice	Not all subgroups meets $p > 0.05$	Not all subgroups meets $p > 0.05$	Friedman's M test	$X^2_{within\ group}$=131.163, df=6	<0.0001	****
								$X^2_{between\ group}$=141.831, df=3	<0.0001	****

**Fig1B**	PWMT(g)	Control-Basal (n=10),<br>PI-Basal (n=10),<br>PI+ST36-Basal (n=10),<br>PI+Sham-ST36 Basal (n=10).<br>Control-1d (n=10),<br>Pl-1d (n=10),<br>PI+ST36-1d (n=10),<br>PI+Sham-ST36-1d (n=10).<br>. Control-2d (n=10),<br>Pl-2d (n=10),<br>PI+ST36-2d (n=10),<br>PI+Sham-ST36-2d (n=10).<br>Control-3d (n=10),<br>Pl-3d (n=10),<br>PI+ST36-3d (n=10),<br>PI+Sham-ST36-3d (n=10).<br>Control-4d (n=10),<br>Pl-4d (n=10),<br>PI+ST36-4d (n=10),<br>PI+Sham-ST36-4d (n=10).<br>Control-5d (n=10),<br>Pl-5d (n=10),<br>PI+ST36-5d (n=10),<br>PI+Sham-ST36-5d (n=10).<br>Control-6d (n=10),<br>Pl-6d (n=10),<br>PI+ST36-6d (n=10),<br>PI+Sham-ST36-6d (n=10).	mouse	40 mice	Not all subgroups meets *p* > 0.05	Not all subgroups meets *p* > 0.05	Friedman's M test	$X^2_{within\ group}$=131.163, df=6	<0.0001	****
								$X^2_{between\ group}$=49.028, df=3	<0.0001	****
		Control-1d vs.Pl-1d					Dunn's multiple comparisons test		<0.0001	****
		Pl-1d vs. PI+ST36-1d							0.0286	*
		Control-2d vs.Pl-2d					Dunn's multiple comparisons test		<0.0001	****
		Pl-2d vs.PI+ST36-2d							0.0062	**

		Control-3d vs.PI-3d					Dunn's multiple comparisons test		<0.0001	****
		PI-3d vs.PI+ST36-3d							0.0066	**
**Fig1C**	PWMT(g)	Control-Basal (n=10),<br>PI-Basal (n=10),<br>PI+ST36-Basal (n=10),<br>PI+Sham-ST36 Basal (n=10).<br>Control-1d (n=10),<br>PI-1d (n=10),<br>PI+ST36-1d (n=10),<br>PI+Sham-ST36-1d (n=10).<br>Control-2d (n=10),<br>PI-2d (n=10),<br>PI+ST36-2d (n=10),<br>PI+Sham-ST36-2d (n=10).<br>Control-3d (n=10),<br>PI-3d (n=10),<br>PI+ST36-3d (n=10),<br>PI+Sham-ST36-3d (n=10).<br>Control-4d (n=10),<br>PI-4d (n=10),<br>PI+ST36-4d (n=10),<br>PI+Sham-ST36-4d (n=10).<br>Control-5d (n=10),<br>PI-5d (n=10),<br>PI+ST36-5d (n=10),<br>PI+Sham-ST36-5d (n=10).<br>Control-6d (n=10),<br>PI-6d (n=10),<br>PI+ST36-6d (n=10),<br>PI+Sham-ST36-6d (n=10).	mouse	40 mice	Not all subgroups meets *p* > 0.05	Not all subgroups meets *p* > 0.05	Friedman's M test	$X^2_{within\ group}$=125.829, df=6	<0.0001	****
								$X^2_{between\ group}$=99.855, df=3	<0.0001	****

**Fig1D**	PWTL(s)	Control-Basal (n=10),<br>PI-Basal (n=10),<br>PI+ST36-Basal (n=10),<br>PI+Sham-ST36 Basal (n=10).<br>Control-1d (n=10),<br>Pl-1d (n=10),<br>PI+ST36-1d (n=10),<br>PI+Sham-ST36-1d (n=10).<br>Control-2d (n=10),<br>Pl-2d (n=10),<br>PI+ST36-2d (n=10),<br>PI+Sham-ST36-2d (n=10).<br>Control-3d (n=10),<br>Pl-3d (n=10),<br>PI+ST36-3d (n=10),<br>PI+Sham-ST36-3d (n=10).<br>Control-4d (n=10),<br>Pl-4d (n=10),<br>PI+ST36-4d (n=10),<br>PI+Sham-ST36-4d (n=10).<br>Control-5d (n=10),<br>Pl-5d (n=10),<br>PI+ST36-5d (n=10),<br>PI+Sham-ST36-5d (n=10).<br>Control-6d (n=10),<br>Pl-6d (n=10),<br>PI+ST36-6d (n=10),<br>PI+Sham-ST36-6d (n=10).	mouse	40 mice	Not all subgroups meets p > 0.05	Not all subgroups meets p > 0.05	Friedman's M test	$X^2_{within\ group}$=36.750, df=3	<0.0001	****
								$X^2_{between\ group}$=51.812, df=3	<0.0001	****
		Control-1d vs.Pl-1d					Dunn's multiple comparisons test		0.0026	**
		Pl-1d vs. PI+ST36-1d							0.7558	ns

		Control-2d vs.PI-2d					Dunn's multiple comparisons test		<0.0001	****
		PI-2d vs.PI+ST36-2d							<0.0001	****
		Control-3d vs.PI-3d					Dunn's multiple comparisons test		<0.0001	****
		PI-3d vs.PI+ST36-3d							0.0012	**
**Fig1E**	PWMT(g)	Control-Basal (n=10),<br>PI-Basal (n=10),<br>PI+BL60 Basal (n=10).<br>Control-1d (n=10),<br>PI-1d (n=10),<br>PI+BL60-1d (n=10). .<br>Control-2d (n=10),<br>PI-2d (n=10),<br>PI+BL60-2d (n=10),<br>Control-3d (n=10),<br>PI-3d (n=10),<br>PI+BL60-3d (n=10),<br>Control-4d (n=10),<br>PI-4d (n=10),<br>PI+BL60-4d (n=10),<br>Control-5d (n=10),<br>PI-5d (n=10),<br>PI+BL60-5d (n=10),<br>Control-6d (n=10),<br>PI-6d (n=10),<br>PI+BL60-6d (n=10).	mouse	30 mice	Not all subgroups meets p > 0.05	Not all subgroups meets p > 0.05	Friedman's M test	$X^2_{within\ group}$=59.428, df=6	<0.0001	****
								$X^2_{between\ group}$=66.341, df=2	<0.0001	****

**Fig1F**	PWMT(g)	Control-Basal (n=10),<br>PI-Basal (n=10),<br>PI+BL60 Basal (n=10).<br>Control-1d (n=10),<br>Pl-1d (n=10),<br>PI+BL60-1d (n=10). .<br>Control-2d (n=10),<br>Pl-2d (n=10),<br>PI+BL60-2d (n=10),<br>Control-3d (n=10),<br>Pl-3d (n=10),<br>PI+BL60-3d (n=10)	mouse	30 mice	Not all subgroups meets *p* > 0.05	Not all subgroups meets *p* > 0.05	Friedman's M test	X^2within group=26.511, df=3	<0.0001	****
								X^2between group=29.020, df=2	<0.0001	****
		Control-1d vs.Pl-1d	mouse	20 mice			Dunn's multiple comparisons test		0.0002	***
		Control-1d vs. PI+BL60-1d							0.0006	***
		Control-2d vs.Pl-2d	mouse	20 mice			Dunn's multiple comparisons test		0.0002	***
		Control-2d vs.PI+BL60-2d							0.0006	***
		Control-3d vs.Pl-3d	mouse	20 mice			Dunn's multiple comparisons test		0.0088	**
		Control-3d vs.PI+BL60-3d							<0.0001	****

**Fig1G**	PWMT(g)	Control-Basal (n=10),<br>PI-Basal (n=10),<br>PI+BL60 Basal (n=10).<br>Control-1d (n=10),<br>PI-1d (n=10),<br>PI+BL60-1d (n=10). .<br>Control-2d (n=10),<br>PI-2d (n=10),<br>PI+BL60-2d (n=10),<br>Control-3d (n=10),<br>PI-3d (n=10),<br>PI+BL60-3d (n=10),<br>Control-4d (n=10),<br>PI-4d (n=10),<br>PI+BL60-4d (n=10),<br>Control-5d (n=10),<br>PI-5d (n=10),<br>PI+BL60-5d (n=10),<br>Control-6d (n=10),<br>PI-6d (n=10),<br>PI+BL60-6d (n=10).	mouse	30 mice	W=0.964 *p* =0.826,<br>W=0.910 *p* =0.2835,<br>W=0.888 *p* =0.162,<br>W=0.861 *p* =0.079,<br>W=0.875 *p* =0.116,<br>W=0.925 *p* =0.405,<br>W=0.905 *p* =0.245,<br>W=0.940 *p* =0.554,<br>W=0.949 *p* =0.655,<br>W=0.936 *p* =0.514<br>W=0.950 *p* =0.667,<br>W=0.862 *p* =0.081,<br>W=0.988 *p* =0.995,<br>W=0.953 *p* =0.706,<br>W=0.966 *p* =0.854,<br>W=0.932 *p* =0.463,<br>W=0.914 *p* =0.313,<br>W=0.987 *p* =0.993,<br>W=0.959 *p* =0.777,<br>W=0.932 *p* =0468,<br>W=0.982 *p* =0.975.	F=0.622, DFn=2,<br>DFd=27, *p* =0.545<br>F=1.247, DFn=2,<br>DFd=27, *p* =0.303<br>F=3.228, DFn=2,<br>DFd=27, *p* =0.055<br>F=0.060, DFn=2,<br>DFd=27, *p* =0.942<br>F=0.043, DFn=2,<br>DFd=27, *p* =0.958<br>F=1.574, DFn=2,<br>DFd=27, *p* =0.226<br>F=0.236, DFn=2,<br>DFd=27, *p* =0.791	Repeated Measures ANOVA	$F_{within\ group}$=60.329, df_1=6,df_2=162	<0.0001	****
								$F_{between\ group}$=95.866, df_1=2,df_2=27	<0.0001	****

**Fig1H**	PWMT(g)	Control-Basal (n=10), PI-Basal (n=10), PI+BL60 Basal (n=10). Control-1d (n=10), Pl-1d (n=10), PI+BL60-1d (n=10). . Control-2d (n=10), Pl-2d (n=10), PI+BL60-2d (n=10), Control-3d (n=10), Pl-3d (n=10), PI+BL60-3d (n=10)	mouse	30 mice	W=0.964 p=0.826, W=0.910 p=0.283, W=0.888 p=0.162, W=0.861 p=0.079, W=0.875 p=0.116, W=0.925 p=0.405, W=0.905 p=0.245, W=0.940 p=0.554, W=0.949 p=0.655, W=0.936 p=0.514 W=0.950 p=0.667, W=0.862 p=0.081,	F=0.622, DFn=2, DFd=27, p=0.545 F=1.247, DFn=2, DFd=27, p=0.303 F=3.228, DFn=2, DFd=27, p=0.055 F=0.060, DFn=2, DFd=27, p=0.942	Repeated Measures ANOVA	$F_{within\ group}$=101.987, df_1=3,df_2=81	<0.0001	****
								$F_{between\ group}$=143.823, df_1=2,df_2=27	<0.0001	****
		Control-1d vs.Pl-1d					Tukey's multiple comparisons test		<0.0001	****
		Control-2d vs.Pl-2d					Tukey's multiple comparisons test		<0.0001	****
		Control-3d vs.Pl-3d					Tukey's multiple comparisons test		<0.0001	****

**Fig1K**	Max Contact Max Intensity	Control (n=10), PI (n=10). PI+ST36 (n=10), PI+Sham-ST36 (n=10).	mouse	40mice	Not all subgroups meets $p > 0.05$		Kruskal - Wallis H test	χ2 =29.32, DF=4	<0.0001	****
		Control vs. PI					Dunn's multiple comparisons test		0.0001	****
		PI vs. PI+ST36					Dunn's multiple comparisons test		0.031	*
		PI+ST36 vs. PI+Sham-ST36					Dunn's multiple comparisons test		0.007	**
**Fig1L**	Paw Area PI+ST36 (cm^2)	Control (n=10), PI (n=10). PI+ST36 (n=10), PI+Sham-ST36 (n=10).	mouse	40mice	W=0.985 p =0.985, W=0.922 p =0.375, W=0.929 p =0.437, W=0.893 p =0.185.	F=0.954, DFn=3, DFd=36, p =0.425	One-way ANOVA	F=17.3, DFn=3, DFd=36.	<0.0001	****
		Control vs. PI					Tukey's multiple comparisons test		<0.0001	****
		PI vs. PI+ST36					Tukey's multiple comparisons test		0.042	*
		PI+ST36 vs. PI+Sham-ST36					Tukey's multiple comparisons test		<0.0001	****

**Fig1M**	Max Contact Area PI+ST36 (cm^2)	Control (n=10),<br>PI (n=10).<br>PI+ST36 (n=10),<br>PI+Sham-ST36 (n=10).	mouse	40mice	W=0.959 *p* =0.778,<br>W=0.952 *p* =0.691,<br>W=0.915 *p* =0.320,<br>W=0.913 *p* =0.300.	F=0.793, DFn=3,<br>DFd=36, *p* =0.506	One-way ANOVA	F=16.67, DFn=3,<br>DFd=36.	<0.0001	****
		Control vs. PI					Tukey's multiple comparisons test		<0.0001	****
		PI vs. PI+ST36					Tukey's multiple comparisons test		0.05	*
		PI+ST36 vs. PI+Sham-ST36					Tukey's multiple comparisons test		0.001	***
**Fig1N**	Max Intensity (PI+ST36)	Control (n=10),<br>PI (n=10).<br>PI+ST36 (n=10),<br>PI+Sham-ST36 (n=10).	mouse	40mice	Not all subgroups meets *p* > 0.05		Kruskal - Wallis H test	χ2 =30.558,<br>DF=3	<0.0001	****
		Control vs. PI					Dunn's multiple comparisons test		<0.0001	****
		PI vs. PI+ST36					Dunn's multiple comparisons test		0.028	*
		PI+ST36 vs. PI+Sham-ST36					Dunn's multiple comparisons test		0.003	**

**Fig1O**	PI+ST36 Intensity of the 15 Most Intense Pixels	Control (n=10), PI (n=10). PI+ST36 (n=10), PI+Sham-ST36 (n=10).	mouse	40mice	W=0.898 *p* =0.209, W=0.980 *p* =0.966, W=0.907 *p* =0.263, W=0.915 *p* =0.314.	F=0.0.165, DFn=3, DFd=36, *p* =0.919	One-way ANOVA	F=50.276, DFn=3, DFd=36.	<0.0001	****
		Control vs. PI					Tukey's multiple comparisons test		<0.0001	****
		PI vs. PI+ST36					Tukey's multiple comparisons test		0.034	*
		PI+ST36 vs. PI+Sham-ST36					Tukey's multiple comparisons test		<0.0001	****
**Fig1Q**	Swelling of the PI Feet (mm)	Control-Control (n=10), PI-Control (n=10), PI+ST36-Control (n=10). Control-2d (n=10), Pl-2d (n=10), PI+ST36-2d (n=10). Control-3d (n=10), Pl-3d (n=10), PI+ST36-3d (n=10),	mouse	30 mice	Not all subgroups meets *p* > 0.05	Not all subgroups meets *p* > 0.05	Friedman's M test	$X^2_{within\ group}$=16.530, df=2	<0.0001	****
								$X^2_{between\ group}$=22.78, df=2	<0.0001	****

**Fig1R**	Swelling of the PI Feet (mm)	Control (n=10), PI (n=10). PI+ST36 (n=10),	mouse	30mice	Not all subgroups meets *p* > 0.05	Not all subgroups meets *p* > 0.05	Kruskal - Wallis H test	χ2 =24.384, DF=2	<0.0001	****
		Control vs. PI					Dunn's multiple comparisons test		<0.0001	****
		PI vs. PI+ST36					Dunn's multiple comparisons test		<0.0001	****
**Fig2B**	c-Fos+ Neurons (n)	Control (n=6), PI (n=6). PI+EA (n=6), PI+Sham-EA (n=6).	mouse	24mice	W=0.905 p=0.407, W=0.851 p=0.161, W=0.911 p=0.446, W=0.858 p=0.183.	F=1.840, DFn=3, DFd=20, p=0.172	One-way ANOVA	F=25.024, DFn=3, DFd=20.	<0.0001	****
		Control vs. PI					Tukey's multiple comparisons test		0.031	*
		PI vs. PI+EA					Tukey's multiple comparisons test		<0.0001	****

**Fig2C**	Merged/c-Fos+ Neurons (n)	Control (n=6),<br>PI (n=6).<br>PI+EA (n=6),<br>PI+Sham-EA (n=6).	mouse	24mice	W=0.948 *p* =0.728,<br>W=0.972 *p* =0.905,<br>W=0.958 *p* =0.804,<br>W=0.865 *p* =0.206.	F=1.999, DFn=3, DFd=20, *p* =0.147	One-way ANOVA	F=18.765, DFn=3, DFd=20.	<0.0001	****
		Control vs. PI					Tukey's multiple comparisons test		0.017	*
		PI vs. PI+EA					Tukey's multiple comparisons test		<0.0001	****
**Fig2F**	AP frequency (Hz）	Control (n=7),<br>PI (n=5).<br>PI+EA (n=7),	Neuron	3 mice per group	Not all subgroups meets *p* > 0.05	Not all subgroups meets *p* > 0.05	Kruskal - Wallis H test	χ2 =6.325, DF=2	0.042	****
		Control vs. PI					Dunn's multiple comparisons test		0.029	**
		PI vs. PI+EA					Dunn's multiple comparisons test		0.019	*
**Fig2H**	Number of spiking	Control (n=10),<br>PI (n=9).<br>PI+EA (n=17)	mouse	3 mice per group	Not all subgroups meets *p* > 0.05	Not all subgroups meets *p* > 0.05	Friedman's M test	X^2=87.425, df=2	<0.0001	****

**Fig2I**	AP rheobase (pA)	Control (n=13), PI (n=16). PI+EA (n=22),	Neuron	3 mice per group	Not all subgroups meets *p* > 0.05	Not all subgroups meets *p* > 0.05	Kruskal - Wallis H test	χ2 =16.295, DF=2	<0.0001	****
		Control vs. PI					Dunn's multiple comparisons test		<0.0001	****
		PI vs. PI+EA					Dunn's multiple comparisons test		0.041	*
**Fig2M**	ΔF/F-AUC	Control (n=4), PI+EA (n=4)	mouse	8 mice	W=0.986 *p* =0.934, W=0.972 *p* =0.855	F=5.720, DFn=1, DFd=6, *p* =0.054	Two-tailed unpaired t test	t=-2.963, DF=6	0.025	*
**Fig2N**	ΔF/F-PI+ST36	Control (n=4), PI+EA (n=4)	mouse	8 mice	W=0.959 *p* =0.773, W=0.977 *p* =0.886	F=2.591, DFn=1, DFd=6, *p* =0.159	Two-tailed unpaired t test	t=-3.058, DF=6	0.022	*

**Fig3D**	PWMT(g)	Control-Basal (n=10),<br>PI-Basal (n=10),<br>PI+EA-Basall (n=10),<br>NpHR+PI+EA-Basal(n=10).<br>Control-1d (n=10),<br>Pl-1d (n=10),<br>PI+EA-1d (n=10),<br>NpHR+PI+EA-1d (n=10).<br>Control-2d (n=10),<br>Pl-2d (n=10),<br>PI+EA-2d (n=10),<br>NpHR+PI+EA-2d (n=10).<br>Control-3d (n=10),<br>Pl-3d (n=10),<br>PI+EA-3d (n=10),<br>NpHR+PI+EA-3d (n=10).	mouse	40 mice	Not all subgroups meets *p* > 0.05	Not all subgroups meets *p* > 0.05	Friedman's M test	$X^2_{within\ group}$=36.985, df=3	<0.0001	****
								$X^2_{between\ group}$=52.844, df=3	<0.0001	****

**Fig3E**	PWMT(g)	Control-Basal (n=10), PI-Basal (n=10), PI+EA-Basall (n=10), NpHR+PI+EA-Basal(n=10). Control-1d (n=10), Pl-1d (n=10), PI+EA-1d (n=10), NpHR+PI+EA-1d (n=10). Control-2d (n=10), Pl-2d (n=10), PI+EA-2d (n=10), NpHR+PI+EA-2d (n=10). Control-3d (n=10), Pl-3d (n=10), PI+EA-3d (n=10), NpHR+PI+EA-3d (n=10).	mouse	40 mice	Not all subgroups meets *p* > 0.05	Not all subgroups meets *p* > 0.05	Friedman's M test	$X^2_{within\ group}$=36.985, df=3	<0.0001	****
								$X^2_{between\ group}$=52.844, df=3	<0.0001	****
		NpHR+PI+EA-1d vs.Pl-1d					Dunn's multiple comparisons test		>0.9999	ns
		PI+EA-1d vs. NpHR+PI+EA-1d							0.1978	ns
		NpHR+PI+EA-2d vs.Pl-2d					Dunn's multiple comparisons test		>0.9999	ns
		PI+EA-2d vs.NpHR+PI+EA-2d							0.043	*
		NpHR+PI+EA-3d vs.Pl-3d					Dunn's multiple comparisons test		>0.9999	ns
		PI+EA-3d vs.NpHR+PI+EA-3d							0.0474	*

**Fig3F**	PWTL(s)	Control-Basal (n=10), PI-Basal (n=10), PI+EA-Basall (n=10), NpHR+PI+EA-Basal(n=10). Control-1d (n=10), Pl-1d (n=10), PI+EA-1d (n=10), NpHR+PI+EA-1d (n=10). Control-2d (n=10), Pl-2d (n=10), PI+EA-2d (n=10), NpHR+PI+EA-2d (n=10). Control-3d (n=10), Pl-3d (n=10), PI+EA-3d (n=10), NpHR+PI+EA-3d (n=10).	mouse	40 mice	Not all subgroups meets p > 0.05	Not all subgroups meets p > 0.05	Friedman's M test	$X^2_{within\ group}$=49.410, df=3	<0.0001	****
								$X^2_{between\ group}$=55.147, df=3	<0.0001	****

**Fig3G**	PWMT(g)	Control-Basal (n=10), PI-Basal (n=10), PI+EA-Basall (n=10), NpHR+PI+EA-Basal(n=10). Control-1d (n=10), Pl-1d (n=10), PI+EA-1d (n=10), NpHR+PI+EA-1d (n=10). Control-2d (n=10), Pl-2d (n=10), PI+EA-2d (n=10), NpHR+PI+EA-2d (n=10). Control-3d (n=10), Pl-3d (n=10), PI+EA-3d (n=10), NpHR+PI+EA-3d (n=10).	mouse	40 mice	Not all subgroups meets *p* > 0.05		Friedman's M test	$X^2_{within\ group}$=49.410, df=3	<0.0001	****
								$X^2_{between\ group}$=55.147, df=3	<0.0001	****
		NpHR+PI+EA-1d vs.Pl-1d					Dunn's multiple comparisons test		0.7278	ns
		PI+EA-1d vs. NpHR+PI+EA-1d							0.6487	ns
		NpHR+PI+EA-2d vs.Pl-2d					Dunn's multiple comparisons test		0.7007	ns
		PI+EA-2d vs. NpHR+PI+EA-2d							0.0479	*
		NpHR+PI+EA-2d vs.Pl-2d					Dunn's multiple comparisons test		0.554	ns
		PI+EA-3d vs.NpHR+PI+EA-3d							0.0374	*

**Fig3J**	PWMT(g)	Control-Basal (n=10),<br>PI-Basal (n=10),<br>PI+EA-Basal (n=10),<br>PI+ChR2 Basal (n=10).<br>Control-1d (n=10),<br>PI-1d (n=10),<br>PI+EA-1d (n=10),<br>PI+ChR2-1d (n=10).<br>Control-2d (n=10),<br>PI-2d (n=10),<br>PI+EA-2d (n=10),<br>PI+ChR2-2d (n=10).<br>Control-3d (n=10),<br>PI-3d (n=10),<br>PI+EA-3d (n=10),<br>PI+ChR2-3d (n=10).	mouse	40 mice	Not all subgroups meets *p* > 0.05	Not all subgroups meets *p* > 0.05	Friedman's M test	$X^2_{within\ group}$=39.458, df=3	<0.0001	****
								$X^2_{between\ group}$=64.530, df=3	<0.0001	****

**Fig3K**	PWMT(g)	Control-Basal (n=10), PI-Basal (n=10), PI+EA-Basal (n=10), PI+ChR2 Basal (n=10). Control-1d (n=10), PI-1d (n=10), PI+EA-1d (n=10), PI+ChR2-1d (n=10). Control-2d (n=10), PI-2d (n=10), PI+EA-2d (n=10), PI+ChR2-2d (n=10). Control-3d (n=10), PI-3d (n=10), PI+EA-3d (n=10), PI+ChR2-3d (n=10).	mouse	40 mice	Not all subgroups meets *p* > 0.05	Not all subgroups meets *p* > 0.05	Friedman's M test	$X^2_{within\ group}$=39.458, df=3	<0.0001	****
								$X^2_{between\ group}$=64.530, df=3	<0.0001	****

		Pl-1d vs.PI+ChR2-1d					Dunn's multiple comparisons test		0.0054	**
		PI+EA-1d vs.PI+ChR2-1d							>0.9999	ns
		Pl-2d vs.PI+ChR2-2d					Dunn's multiple comparisons test		0.0025	**
		PI+EA-2d vs.PI+ChR2-2d							>0.9999	ns
		Pl-3d vs.PI+ChR2-3d					Dunn's multiple comparisons test		0.0003	***
		PI+EA-3d vs.PI+ChR2-3d							0.2062	ns
**Fig3L**	PWTL(s)	Control-Basal (n=10),<br>PI-Basal (n=10),<br>PI+EA-Basal (n=10),<br>PI+ChR2 Basal (n=10).<br>Control-1d (n=10),<br>Pl-1d (n=10),<br>PI+EA-1d (n=10),<br>PI+ChR2-1d (n=10).<br>Control-2d (n=10),<br>Pl-2d (n=10),<br>PI+EA-2d (n=10),<br>PI+ChR2-2d (n=10).<br>Control-3d (n=10),<br>Pl-3d (n=10),<br>PI+EA-3d (n=10),<br>PI+ChR2-3d (n=10).	mouse	40 mice	Not all subgroups meets *p* > 0.05	Not all subgroups meets *p* > 0.05	Friedman's M test	$X^2_{within\ group}$=43.380, df=3	<0.0001	****
								$X^2_{between\ group}$=52.770, df=3	<0.0001	****

**Fig3M**	PWMT(g)	Control-Basal (n=10),<br>PI-Basal (n=10),<br>PI+EA-Basal (n=10),<br>PI+ChR2 Basal (n=10).<br>Control-1d (n=10),<br>PI-1d (n=10),<br>PI+EA-1d (n=10),<br>PI+ChR2-1d (n=10).<br>Control-2d (n=10),<br>PI-2d (n=10),<br>PI+EA-2d (n=10),<br>PI+ChR2-2d (n=10).<br>Control-3d (n=10),<br>PI-3d (n=10),<br>PI+EA-3d (n=10),<br>PI+ChR2-3d (n=10).	mouse	40 mice	Not all subgroups meets *p* > 0.05	Not all subgroups meets *p* > 0.05	Friedman's M test	$X^2_{within\ group}$=43.380, df=3	<0.0001	****
								$X^2_{between\ group}$=52.770, df=3	<0.0001	****
		PI-1d vs.PI+ChR2-1d					Dunn's multiple comparisons test		0.0026	**
		PI+EA-1d vs.PI+ChR2-1d							>0.9999	ns
		PI-2d vs.PI+ChR2-2d					Dunn's multiple comparisons test		0.0001	***
		PI+EA-2d vs.PI+ChR2-2d							0.5999	ns
		PI-3d vs.PI+ChR2-3d					Dunn's multiple comparisons test		<0.0001	****
		PI+ST36-3d vs.PI+ChR2-3d							0.9085	ns

**Fig4A**	NE in Serum (ng/ml)	Control (n=3),<br>PI (n=3).<br>PI+EA (n=3),<br>PI+Sham-EA (n=3).	Tissue homogenization	6 mice per group	W=0.7875 *p* =0.081,<br>W=0.967 *p* =0.823,<br>W=0.957 *p* =0.759,<br>W=0.945 *p* =0.682.	F=1.275, DFn=3, DFd=12, *p* =0.327	One-way ANOVA	F=1.141, DFn=3, DFd=12.	0.372	ns
**Fig4B**	NE in SDH (ng/ml)	Control (n=3),<br>PI (n=3).	Tissue homogenization	6 mice per group	W=0.999 *p* =0.953,<br>W=0.961 *p* =0.619,	F=1.035, DFn=2, DFd=4, *p* =0.367	Two-tailed unpaired t test	t=5.105, DF=4	0.007	**
**Fig4C**	NE in Serum (ng/ml)	PI (n=3).<br>PI+EA (n=3),<br>PI+Sham-EA (n=3).	Tissue homogenization	6 mice per group	W=0.961 *p* =0.619,<br>W=0.986 *p* =0.770,<br>W=0.816 *p* =0.152.	F=0.3956, DFn=2, DFd=6, *p* =0.6897	One-way ANOVA	F=10.109, DFn=2, DFd=6.	0.012	*
		PI+Sham-EA vs. PI+EA					Tukey's multiple comparisons test		0.0178	*
		PI vs. PI+EA					Tukey's multiple comparisons test		0.0201	*
**Fig4E**	Δ F/F	Control (n=18),<br>PI+Sham-EA (n=18).	Neuron	3 mice per group	W=0.806 *p* =0.002<br>W=0.784 *p* =0.001.	F=0.083, DFn=1, DFd=34, *p* =0.775	Mann-Whitney U test		0.359	ns

**Fig4F**	Δ F/F	Control (n=18),<br>PI+EA (n=18).	Neuron	3 mice per group	W=0.793 p =0.001<br>W=0.880 p =0.026.	F=2.497, DFn=1,<br>DFd=34, p =0.123	Mann-Whitney U test		<0.0001	****
**Fig4G**	Δ F/F	Control (n=18),<br>PI+ChR2 (n=18).	Neuron	3 mice per group	W=0.797 p =0.001<br>W=0.774 p =0.001.	F=5.290, DFn=1,<br>DFd=34, p =0.028	Mann-Whitney U test		0.001	**
**Fig4H**	Δ F/F	Control (n=18),<br>PI+NpHR+EA (n=18).	Neuron	3 mice per group	W=0.924 p =0.150<br>W=0.843 p =0.007.	F=0.936, DFn=1,<br>DFd=34, p =0.340	Mann-Whitney U test		<0.0001	****
**Fig5D**	Δ F/F-PI+ST36	Control (n=24),<br>PI (n=24).<br>PI+EA (n=24),	Neuron response	3 mice per group	Not all subgroups meets p > 0.05	Not all subgroups meets p > 0.05	Kruskal - Wallis H test	χ2 =43.872, DF=2	<0.0001	****
		Control vs. PI					Dunn's multiple comparisons test		<0.0001	****
		PI vs. PI+EA					Dunn's multiple comparisons test		<0.0001	****

**Fig5E**	Δ F/F-AUC	Control (n=24), PI (n=24). PI+EA (n=24),	Neuron response	3 mice per group	Not all subgroups meets *p* > 0.05	Not all subgroups meets *p* > 0.05	Kruskal - Wallis H test	χ2 =41.653, DF=2	<0.0001	****
		Control vs. PI					Dunn's multiple comparisons test		<0.0001	****
		PI vs. PI+EA					Dunn's multiple comparisons test		<0.0001	****
**Fig5H**	Δ F/F-PI+ST36	Control (n=9), PI (n=9). PI+EA (n=9),	Neuron response	3 mice per group	Not all subgroups meets *p* > 0.05	Not all subgroups meets *p* > 0.05	Kruskal - Wallis H test	χ2 =15.728, DF=2	<0.0001	****
		Control vs. PI					Dunn's multiple comparisons test		<0.0001	****
		PI vs. PI+EA					Dunn's multiple comparisons test		0.022	*
**Fig5I**	Δ F/F-AUC	Control (n=9), PI (n=9). PI+EA (n=9),	Neuron response	3 mice per group	Not all subgroups meets *p* > 0.05	Not all subgroups meets *p* > 0.05	Kruskal - Wallis H test	χ2 =17.252, DF=2	<0.0001	****
		Control vs. PI					Dunn's multiple comparisons test		<0.0001	****
		PI vs. PI+EA					Dunn's multiple comparisons test		0.014	*

**Fig5N**	Δ F/F-PI+ST36	Control (n=24),<br>PI (n=24).<br>PI+EA (n=24),	Neuron	3 mice per group	Not all subgroups meets *p* > 0.05	Not all subgroups meets *p* > 0.05	Kruskal - Wallis H test	F=19.35, DFn=3, DFd=27.	<0.0001	****
		Control vs. PI					Dunn's multiple comparisons test		0.001	***
		PI vs. PI+EA					Dunn's multiple comparisons test		<0.0001	****
**Fig5O**	Δ F/F-AUC	Control (n=24),<br>PI (n=24).<br>PI+EA (n=24),	Neuron	3 mice per group	Not all subgroups meets *p* > 0.05	Not all subgroups meets *p* > 0.05	Kruskal - Wallis H test	F=11.672, DFn=2, DFd=93.	<0.0001	****
		Control vs. PI					Dunn's multiple comparisons test		0.0034	**
		PI vs. PI+ST36					Dunn's multiple comparisons test		<0.0001	****
**Fig6B**	α_{2A}-AR / GAPDH	Control (n=3),<br>PI (n=3).	Tissue homogeniz ation	6 mice per group	W=0.81 *p* =0.138<br>W=0.962 *p* =0.625.	F=5.854, DFn=1, DFd=4, *p* =0.073	Two-tailed unpaired t test	t=4.492 DF=4	0.011	*

**Fig6C**	α_{2A}-AR / GAPDH	Control (n=4),<br>PI (n=4).<br>PI+EA (n=4),	Tissue homogenization	8 mice per group	W=0.970 *p* =0.844<br>W=0.990 *p* =0.958,<br>W=0.946 *p* =0.688	F=0.375, DFn=2, DFd=9, *p* =0.697	One-way ANOVA	F=74.229, DFn=2, DFd=9.	<0.0001	****
		PI vs. PI+EA					Tukey's multiple comparisons test		<0.0001	****
**Fig6E**	α_{2A}-AR fluorescence intensity	Control (n=6),<br>PI (n=6).<br>PI+EA (n=6),<br>PI+Sham-EA (n=6).	Spinal cord slice	3 mice per group	W=0.906 *p* =0.411,<br>W=0.906 *p* =0.409,<br>W=0.988 *p* =0.982,<br>W=0.962 *p* =0.837.	F=2.550, DFn=3, DFd=20, *p* =0.085	One-way ANOVA	F=110.585, DFn=3, DFd=20.	<0.0001	****
		Control vs. PI					Tukey's multiple comparisons test		<0.0001	****
		Control vs. PI+Sham-EA					Tukey's multiple comparisons test		<0.0001	****
		PI+Sham-EA vs. PI+EA					Tukey's multiple comparisons test		0.002	**

**Fig6F**	p-CaMKII / CaMKII	Control (n=6),<br>PI (n=6).<br>PI+EA (n=6),<br>PI+Sham-EA (n=6).	Tissue homogenization	12 mice per group	W=0.873 *p* =0.237,<br>W=0.970 *p* =0.893,<br>W=0.935 *p* =0.617,<br>W=0.901 *p* =0.382.	F=0.334, DFn=3, DFd=20, *p* =0.801	One-way ANOVA	F=32.671, DFn=3, DFd=20.	<0.0001	****
		Control vs. PI					Tukey's multiple comparisons test		<0.0001	****
		PI vs. PI+EA					Tukey's multiple comparisons test		<0.0001	****
**Fig6H**	p-CaMKII / CaMKII	Control (n=5),<br>PI (n=5).<br>PI+EA (n=5),<br>PI+Sham-EA (n=5).	Tissue homogenization	5 mice per group	W=0.893 *p* =0.372,<br>W=0.887 *p* =0.343,<br>W=0.921 *p* =0.539,<br>W=0.977 *p* =0.918.	F=2.643, DFn=3, DFd=16, *p* =0.085	One-way ANOVA	F=1.008, DFn=3, DFd=16.	0.415	ns

**Fig6J**	p-CaMKII fluorescence intensity	Control (n=3),<br>PI (n=3).<br>PI+EA (n=3),<br>PI+Sham-EA (n=3).	mouse	3 mice per group	W=0.998 *p* =0.92,<br>W=0.897 *p* =0.376,<br>W=0.98 *p* =0.727,<br>W=0.822 *p* =0.169.	F=0.1971, DFn=3, DFd=8, *p* =0.8955	One-way ANOVA	F=12.627, DFn=3, DFd=8.	0.0021	**
		Control vs. PI					Tukey's multiple comparisons test		0.0057	**
		PI+Sham-EA vs. PI+EA					Tukey's multiple comparisons test		0.0217	*
		PI vs. PI+EA					Tukey's multiple comparisons test		0.0127	*
**Fig7B**	p-CaMKII / CaMKII	Control (n=6),<br>PI (n=6).<br>PI+EA (n=6),<br>Yohimbine+PI+EA (n=6).	Tissue homogenization	12 mice per group	W=0.915 *p* =0.468,<br>W=0.839 *p* =0.128,<br>W=0.978 *p* =0.938,<br>W=0.945 *p* =0.699.	F=1.162, DFn=3, DFd=20, *p* =0.349	One-way ANOVA	F=6.728, DFn=3, DFd=20.	0.0026	**
		PI vs. PI+EA					Dunnett's multiple comparisons test		0.0392	*
		Yohimbine+PI+EA vs. PI+EA					Dunnett's multiple comparisons test		0.0345	*

**Fig7E**	Δ F/F-PI+ST36	Control (n=24), PI (n=24). PI+EA (n=24),	Neuron response	3 mice per group	Not all subgroups meets *p* > 0.05	Not all subgroups meets *p* > 0.05	Kruskal - Wallis H test	χ2 =49.36 DF=3	<0.0001	****
		PI vs. PI+EA					Dunn's multiple comparisons test		<0.0001	****
		Yohimbine+PI+EA vs. PI+EA					Dunn's multiple comparisons test		<0.0001	****
**Fig7F**	Δ F/F-AUC	Control (n=27), PI (n=27). PI+EA (n=27),	Neuron response	3 mice per group	Not all subgroups meets *p* > 0.05	Not all subgroups meets *p* > 0.05	Kruskal - Wallis H test	χ2 =50.93, DF=3	<0.0001	****
		PI vs. PI+EA					Dunn's multiple comparisons test		<0.0001	****
		Yohimbine+PI+EAvs. PI+EA					Dunn's multiple comparisons test		<0.0001	****

**Fig7H**	PWMT(g)	Control (n=10),<br>PI (n=10).<br>PI+EA (n=10),<br>Yohimbine+PI+EA (n=10).	mouse	40 mice	Not all subgroups meets *p* > 0.05	Not all subgroups meets *p* > 0.05	Kruskal - Wallis H test	χ2 =33.02, DF=4	<0.0001	****
		PI vs. Control					Dunn's multiple comparisons test		<0.0001	****
		PI vs. PI+EA				`	Dunn's multiple comparisons test		0.0351	*
		Yohimbine+PI+EA vs. PI+EA					Dunn's multiple comparisons test		0.017	*
**Fig7J**	PWTL(s)	Control (n=10),<br>PI (n=10).<br>PI+EA (n=10),<br>Yohimbine+PI+EA (n=10).	mouse	40 mice	W=0.978 *p* =0.951,<br>W=0.965 *p* =0.845,<br>W=0.931 *p* =0.460,<br>W=0.967 *p* =0.866.	F=1.691, DFn=3, DFd=36, *p* =0.186	One-way ANOVA	F=47.3, DFn=3, DFd=36.	<0.0001	****
		PI vs. PI+EA					Tukey's multiple comparisons test		0.0028	**
		Yohimbine+PI+EA vs. PI+EA					Tukey's multiple comparisons test		0.0003	***

**SFig1C**	PWMT(g)	Control-Basal (n=10),<br>PI-Basal (n=10),<br>Control-1d (n=10),<br>Pl-1d (n=10),<br>Control-2d (n=10),<br>PI-2d (n=10),<br>Control-3d (n=10),<br>Pl-3d (n=10),<br>Control-4d (n=10),<br>Pl-4d (n=10),<br>Control-5d (n=10),<br>Pl-5d (n=10),<br>Control-6d (n=10),<br>Pl-6d (n=10),	mouse	20 mice	Not all subgroups meets *p* > 0.05	Not all subgroups meets *p* > 0.05	Friedman's M test	$X^2_{within\ group}$=20.194, df=6	0.003	**
								$X^2_{between\ group}$=40.710, df=1	<0.0001	****

**SFig1D**	PWTL(S)	Control-Basal (n=10),<br>PI-Basal (n=10),<br>Control-1d (n=10),<br>PI-1d (n=10),<br>Control-2d (n=10),<br>PI-2d (n=10),<br>Control-3d (n=10),<br>PI-3d (n=10),<br>Control-4d (n=10),<br>PI-4d (n=10),<br>Control-5d (n=10),<br>PI-5d (n=10),<br>Control-6d (n=10),<br>PI-6d (n=10),	mouse	20 mice	Not all subgroups meets *p* > 0.05	Not all subgroups meets *p* > 0.05	Friedman's M test	$X^2_{within\ group}$=39.300, df=6	<0.0001	****
								$X^2_{between\ group}$=41.657, df=1	<0.0001	****
		LC-PI+EA (n=3),<br>LC-PI+Sham-EA (n=3).				F=1.391, DFn=2, DFd=4, *p* =0.304	Two-tailed unpaired t test	t=-2.851, df=4,	0.046	*
		ACC-PI+EA (n=3),<br>ACC-PI+Sham-EA (n=3).				F=1.638, DFn=2, DFd=4, *p* =0.270	Two-tailed unpaired t test	t=-1.278, df=4,	0.27	ns
		M2-PI+EA (n=3),<br>M2-PI+Sham-EA (n=3).				F=3.818, DFn=2, DFd=4, *p* =0.108	Two-tailed unpaired t test	t=-3.383, df=5,	0.02	**
		M1-PI+EA (n=3),<br>M1-PI+Sham-EA (n=3).				F=10.348, DFn=2, DFd=4, *p* =0.032	Two-tailed unpaired separate variance estimation t test	t=-7.414, df=2.105,	0.015	**
		S1-PI+EA (n=3),<br>S1-PI+Sham-EA (n=3).				F=7.863, DFn=2, DFd=4, *p* =0.038	Two-tailed unpaired separate variance estimation t test	t=3.128, df=4.799,	0.028	**
		S2-PI+EA (n=3),<br>S2-PI+Sham-EA (n=3).				F=0.520, DFn=2, DFd=4, *p* =0.511	Two-tailed unpaired t test	t=0.234, df=4,	0.826	ns

**SFig2B**	c-Fos+ Neurons	IC-PI+EA (n=3), IC-PI+Sham-EA (n=3).	mouse	6mice	All subgroups meets $p > 0.05$	F=0.013, DFn=2, DFd=4, p =0.914	Two-tailed unpaired t test	t=-0.298, df=4,	0.781	ns
		PIR-PI+EA (n=3), PIR-PI+Sham-EA (n=3).				F=0.245, DFn=2, DFd=4, p =0.646	Two-tailed unpaired t-test	t=-7.567, df=4,	0.002	***
		LS-PI+EA (n=3), LS-PI+Sham-EA (n=3).				F=7.002, DFn=2, DFd=4, p =0.057	Two-tailed unpaired t test	t=-2.514, df=4,	0.066	ns
		DB-PI+EA (n=3), DB-PI+Sham-EA (n=3).				F=11.337, DFn=2, DFd=4, p =0.028	Two-tailed unpaired separate variance estimation t test	t=-2.493, df=2.070,	0.126	ns
		V1+V2-PI+EA (n=3), V1+V2-PI+Sham-EA (n=3).				F=1.913, DFn=2, DFd=4, p =0.239	Two-tailed unpaired t test	t=-2.424, df=4,	0.072	ns
		Au-PI+EA (n=3), Au-PI+Sham-EA (n=3).				F=2.467, DFn=2, DFd=4, p =0.191	Two-tailed unpaired t test	t=-1.943, df=4,	0.124	ns
		VTA-PI+EA (n=3), VTA-PI+Sham-EA (n=3).				F=0.104, DFn=2, DFd=4, p =0.763	Two-tailed unpaired t test	t=-1.962, df=4,	0.121	ns
		Amy-PI+EA (n=3), Amy-PI+Sham-EA (n=3).				F=5.388, DFn=2, DFd=4, p =0.081	Two-tailed unpaired t test	t=-3.630, df=4,	0.022	**
		ZI-PI+EA (n=3), ZI-PI+Sham-EA (n=3).				F=10.028, DFn=2, DFd=4, p =0.034	Two-tailed unpaired separate variance estimation t test	t=-2.684, df=2.070,	0.111	ns
		hypothalamus-PI+EA (n=3), hypothalamus-PI+Sham-EA (n=3).				F=12.568, DFn=2, DFd=4, p =0.024	Two-tailed unpaired separate variance estimation t test	t=-2.372, df=2.009,	0.14	ns

		PH-PI+EA (n=3),<br>PH-PI+Sham-EA (n=3).				F=0.319, DFn=2,<br>DFd=4, *p* =0.602	Two-tailed unpaired t test	t=-4.778, df=4,	0.009	***
		thalamus-PI+EA (n=3),<br>thalamus-PI+Sham-EA (n=3).				F=0.057, DFn=2,<br>DFd=4, *p* =0.823	Two-tailed unpaired t test	t=-4.905, df=4,	0.008	***
		SC-PI+EA (n=3),<br>SC-PI+Sham-EA (n=3).				F=0.329, DFn=2,<br>DFd=4, *p* =0.597	Two-tailed unpaired t-test	t=-0.991, df=4,	0.378	ns
		PAG-PI+EA (n=3),<br>PAG-PI+Sham-EA (n=3).				F=5.501, DFn=2,<br>DFd=4, *p* =0.079	Two-tailed unpaired t test	t=-3.427, df=4,	0.027	**
		MNR-PI+EA (n=3),<br>MNR-PI+Sham-EA (n=3).				F=0.020, DFn=2,<br>DFd=4, *p* =0.894	Two-tailed unpaired t test	t=-6.093, df=4,	0.004	***
		RN-PI+EA (n=3),<br>RN-PI+Sham-EA (n=3).				F=0.510, DFn=2,<br>DFd=4, *p* =0.515	Two-tailed unpaired t test	t=-0.985, df=4,	0.38	ns
		RVM-PI+EA (n=3),<br>RVM-PI+Sham-EA (n=3).				F=0.020, DFn=2,<br>DFd=4, *p* =0.896	Two-tailed unpaired t test	t=-7.408, df=4,	0.002	***
		Ect+PRh+Lent-PI+EA (n=3),<br>Ect+PRh+Lent-PI+Sham-EA (n=3).				F=7.591, DFn=2,<br>DFd=4, *p* =0.051	Two-tailed unpaired t test	t=-2.062, df=4,	0.108	ns

**SFig2C**	AP threshold	Control (n=14),<br>PI (n=14).<br>PI+EA (n=20).	Neurons	3 mice per group	Not all subgroups meets *p* > 0.05	Not all subgroups meets *p* > 0.05	Kruskal - Wallis H test	χ2 =11.97, DF=2	0.0025	***
		Control vs. PI					Dunn's multiple comparisons test		0.0488	*
		PI vs. PI+EA					Dunn's multiple comparisons test		0.0021	**
**SFig2D**	AP amplitude	Control (n=13),<br>PI (n=16).<br>PI+EA (n=22).	Neurons	3 mice per group	W=0.936 *p* =0.407,<br>W=0.907 *p* =0.103,<br>W=0.930 *p* =0.122.	F=1.638, DFn=2, DFd=48, *p* =0.205	Kruskal - Wallis H test	χ2 =2.358, DF=2	0.3076	ns
		Control vs. PI					Dunn's multiple comparisons test		0.4833	ns
		PI vs. PI+EA					Dunn's multiple comparisons test		0.6296	ns
**SFig2E**	Half-width	Control (n=13),<br>PI (n=16).<br>PI+EA (n=12).	Neurons	3 mice per group	Not all subgroups meets *p* > 0.05	Not all subgroups meets *p* > 0.05	Kruskal - Wallis H test	χ2 =2.358, DF=2	0.3076	ns
		Control vs. PI					Dunn's multiple comparisons test		0.4833	ns
		PI vs. PI+EA					Dunn's multiple comparisons test		0.6296	ns

**SFig2F**	RMP	Control (n=11),<br>PI (n=15).<br>PI+EA (n=22).	Neurons	3 mice per group	W=0.876 *p* =0.094,<br>W=945 *p* =0.417,<br>W=0.960 *p* =0.492.	F=0.697, DFn=2,<br>DFd=45, *p* =0.503	One-way ANOVA	F=2.915, DF=2	0.0645	ns
		Control vs. PI					Tukey's multiple comparisons test		0.0651	ns
		PI vs. PI+EA					Tukey's multiple comparisons test		0.19	ns
**SFig3B**	PWMT(g)	Control-Basal (n=10),<br>PI-Basal (n=10),<br>mCherry+PI+EA-Basal (n=10).<br>Control-1d (n=10),<br>PI-1d (n=8),<br>mCherry+PI+EA-1d (n=8).<br>Control-2d (n=10),<br>PI-2d (n=8)<br>mCherry+PI+EA-2d (n=8),<br>Control-3d (n=10),<br>PI-3d (n=8),<br>mCherry+PI+EA-3d (n=8),	mouse	26 mice	Not all subgroups meets *p* > 0.05	Not all subgroups meets *p* > 0.05	Friedman's M test	$X^2_{within\ group}$=35.612, df=3	<0.0001	****
								$X^2_{between\ group}$=41.970, df=2	<0.0001	****

**SFig3C**	PWMT(g)	Control-Basal (n=10), PI-Basal (n=10), mCherry+PI+EA-Basal (n=10). Control-1d (n=10), PI-1d (n=8), mCherry+PI+EA-1d (n=8). Control-2d (n=10), PI-2d (n=8) mCherry+PI+EA-2d (n=8), Control-3d (n=10), PI-3d (n=8), mCherry+PI+EA-3d (n=8),	mouse	26 mice	Not all subgroups meets *p* > 0.05	Not all subgroups meets *p* > 0.05	Friedman's M test	$X^2_{within\ group}$=35.612, df=3	<0.0001	****
								$X^2_{between\ group}$=41.970, df=2	<0.0001	****
		Control-1d vs.PI-1d	mouse	26 mice	Not all subgroups meets *p* > 0.05		Dunn's multiple comparisons test		<0.0001	****
		PI-1d vs. mCherry+PI+EA-1d							0.0724	ns
		Control-2d vs.PI-2d	mouse	26 mice	Not all subgroups meets *p* > 0.05		Dunn's multiple comparisons test		<0.0001	****
		PI-2d vs. mCherry+PI+EA-2d							0.0072	**
		Control-3d vs.PI-3d	mouse	26 mice	Not all subgroups meets *p* > 0.05		Dunn's multiple comparisons test		<0.0001	****
		PI-3d vs. mCherry+PI+EA-3d							0.1081	ns

**SFig3E**	PWTL(S)	Control-Basal (n=10), PI-Basal (n=10), mCherry+PI+EA-Basal (n=10). Control-1d (n=10), Pl-1d (n=8), mCherry+PI+EA-1d (n=8). Control-2d (n=10), Pl-2d (n=8) mCherry+PI+EA-2d (n=8), Control-3d (n=10), Pl-3d (n=8), mCherry+PI+EA-3d (n=8),	mouse	26 mice	Not all subgroups meets p > 0.05	Not all subgroups meets p > 0.05	Friedman's M test	$X^2_{within\ group}$=16.477, df=3	0.001	***
								$X^2_{between\ group}$=25.941, df=2	<0.0001	****
**SFig3F**	PWTL(S)	Control-Basal (n=10), PI-Basal (n=10), mCherry+PI+EA-Basal (n=10). Control-1d (n=10), Pl-1d (n=8), mCherry+PI+EA-1d (n=8). Control-2d (n=10), Pl-2d (n=8) mCherry+PI+EA-2d (n=8), Control-3d (n=10), Pl-3d (n=8), mCherry+PI+EA-3d (n=8),	mouse	26 mice	Not all subgroups meets p > 0.05		Friedman's M test	$X^2_{within\ group}$=16.477, df=3	0.001	***
								$X^2_{between\ group}$=25.941, df=2	<0.0001	****

		Control-1d vs.PI-1d	mouse	26 mice	Not all subgroups meets p > 0.05		Dunn's multiple comparisons test		<0.0001	****
		PI-1d vs. mCherry+PI+EA-1d							0.1093	ns
		Control-2d vs.PI-2d	mouse	26 mice	Not all subgroups meets p > 0.05		Dunn's multiple comparisons test		<0.0001	****
		PI-2d vs. mCherry+PI+EA-2d							0.093	ns
		Control-3d vs.PI-3d	mouse	26 mice	Not all subgroups meets p > 0.05		Dunn's multiple comparisons test		0.0002	***
		PI-3d vs. mCherry+PI+EA-3d							0.0098	**
**SFig3H**	PWMT(g)	Control-Basal (n=10),<br>PI-Basal (n=10),<br>PI+mCherry-Basal (n=10).<br>Control-1d (n=10),<br>PI-1d (n=10),<br>PI+mCherry-1d (n=9). .<br>Control-2d (n=10),<br>PI-2d (n=10)<br>PI+mCherry-2d (n=9),<br>Control-3d (n=10),<br>PI-3d (n=10),<br>PI+mCherry-3d (n=9),	mouse	29 mice	Not all subgroups meets p > 0.05	Not all subgroups meets p > 0.05	Friedman's M test	$X^2_{within\ group}$=14.667, df=3	0.002	***
								$X^2_{between\ group}$=22.426, df=2	<0.0001	****

**SFig3I**	PWMT(g)	Control-Basal (n=10),<br>PI-Basal (n=10),<br>PI+mCherry-Basal (n=10).<br>Control-1d (n=10),<br>PI-1d (n=10),<br>PI+mCherry-1d (n=9). .<br>Control-2d (n=10),<br>PI-2d (n=10)<br>PI+mCherry-2d (n=9),<br>Control-3d (n=10),<br>PI-3d (n=10),<br>PI+mCherry-3d (n=9),	mouse	29 mice	Not all subgroups meets $p > 0.05$	Not all subgroups meets $p > 0.05$	Friedman's M test	$X^2_{within\ group}$=14.667, df=3	0.002	***
								$X^2_{between\ group}$=22.426, df=2	<0.0001	****
		Control-1d vs.PI-1d	mouse	29 mice			Dunn's multiple comparisons test		0.0009	***
		Control-1d vs PI+.mCherry -1d							<0.0001	****
		Control-2d vs.PI-2d	mouse	29 mice			Dunn's multiple comparisons test		0.0001	***
		Control-2d vs.PI+mCherry-2d							0.0009	***
		Control-3d vs.PI-3d	mouse	29 mice			Dunn's multiple comparisons test		0.0009	***
		Control-3d vs PI+mCherry-3d							0.0002	***

**SFig3K**	PWTL(S)	Control-Basal (n=10),<br>PI-Basal (n=10),<br>PI+mCherry-Basal (n=10).<br>Control-1d (n=10),<br>PI-1d (n=10),<br>PI+mCherry-1d (n=9). .<br>Control-2d (n=10),<br>PI-2d (n=10)<br>PI+mCherry-2d (n=9),<br>Control-3d (n=10),<br>PI-3d (n=10),<br>PI+mCherry-3d (n=9),	mouse	29mice	W=0.883 *p* =0.140,<br>W=0.934 *p* =0.520,<br>W=0.950 *p* =0.665,<br>W=0.928 *p* =0.431,<br>W=0.960 *p* =0.802,<br>W=0.872 *p* =0.105,<br>W=0.968 *p* =0.876,<br>W=0.914 *p* =0.345,<br>W=0.970 *p* =0.888,<br>W=0.882 *p* =0.139<br>W=0.917 *p* =0.365,<br>W=0.881 *p* =0.133.	Not all subgroups meets $p > 0.05$	Friedman's M test	$X^2_{within\ group}$=32.214, df=3	<0.0001	****
								$X^2_{between\ group}$=23.081, df=2	<0.0001	****
**SFig3L**	PWTL(S)	Control-Basal (n=10),<br>PI-Basal (n=10),<br>PI+mCherry-Basal (n=10),<br>Control-1d (n=10),<br>PI-1d (n=10),<br>PI+mCherry-1d (n=9),<br>Control-2d (n=10),<br>PI-2d (n=10),<br>PI+mCherry-2d (n=9),<br>Control-3d (n=10),<br>PI-3d (n=10),<br>PI+mCherry-3d (n=9),	mouse	29 mice	W=0.883 *p* =0.140,<br>W=0.934 *p* =0.520,<br>W=0.950 *p* =0.665,<br>W=0.928 *p* =0.431,<br>W=0.960 *p* =0.802,<br>W=0.872 *p* =0.105,<br>W=0.968 *p* =0.876,<br>W=0.914 *p* =0.345,<br>W=0.970 *p* =0.888,<br>W=0.882 *p* =0.139<br>W=0.917 *p* =0.365,<br>W=0.881 *p* =0.133.	Not all subgroups meets $p > 0.05$	Friedman's M test	$X^2_{within\ group}$=32.214, df=3	<0.0001	****
								$X^2_{between\ group}$=23.081, df=2	<0.0001	****

		Control-1d vs.PI-1d	mouse	29 mice			Dunn's multiple comparisons test		0.0002	***
		Control-1d vs.PI+mCherry -1d							0.0015	**
		Control-2d vs.PI-2d	mouse	29 mice			Dunn's multiple comparisons test		<0.0001	****
		Control-2d vs.PI+mCherry-2d							0.0046	**
		Control-3d vs.PI-3d	mouse	29 mice			Dunn's multiple comparisons test		<0.0001	****
		Control-3d vs.PI+mCherry-3d							<0.0001	****
**SFig4A**	α_{1A}-AR / GAPDH	Control (n=6), PI (n=6). PI+Sham-PI+EA (n=6), PI+PI+EA (n=6).	Tissue homogenization	12 mice per group	W=0.861 *p* =0.191, W=0.877 *p* =0.255, W=0.934 *p* =0.611, W=0.894 *p* =0.337.	F=0.9320, DFn=3, DFd=20, *p* =0.4436	One-way ANOVA	F=0.4430, DFn=3, DFd=20.	0.7248	ns
**SFig4B**	α_{1B}-AR / GAPDH	Control (n=6), PI (n=6). PI+Sham-PI+EA (n=6), PI+PI+EA (n=6).	Tissue homogenization	12 mice per group	Not all subgroups meets p > 0.05	Not all subgroups meets p > 0.05	Kruskal - Wallis H test	χ2 =2.280, DF=3	0.5164	ns
**SFig4C**	β_2-AR / GAPDH	Control (n=6), PI (n=6). PI+Sham-PI+EA (n=6), PI+PI+EA (n=6).	Tissue homogenization	12 mice per group	Not all subgroups meets p > 0.05	Not all subgroups meets p > 0.05	Kruskal - Wallis H test	χ2 =3.327, DF=3	0.3439	ns

Full blot

Fig 6

B

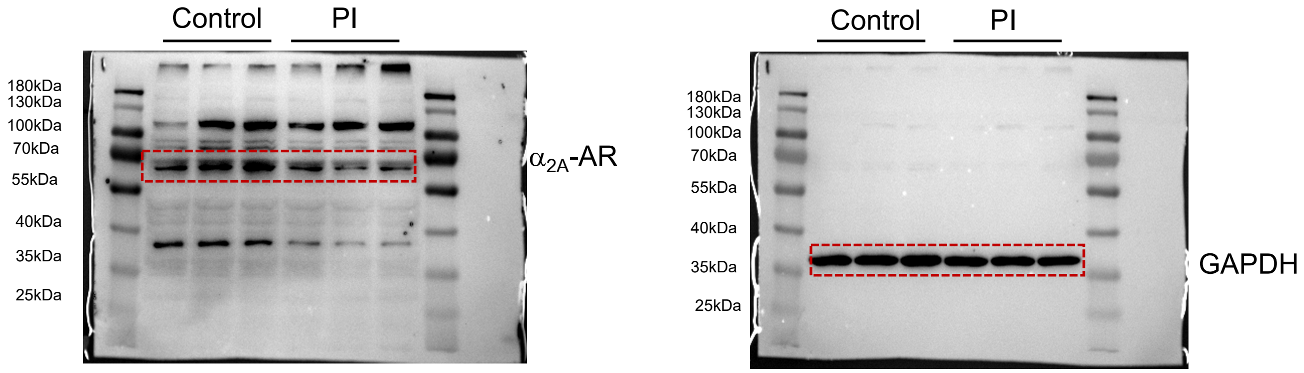

C

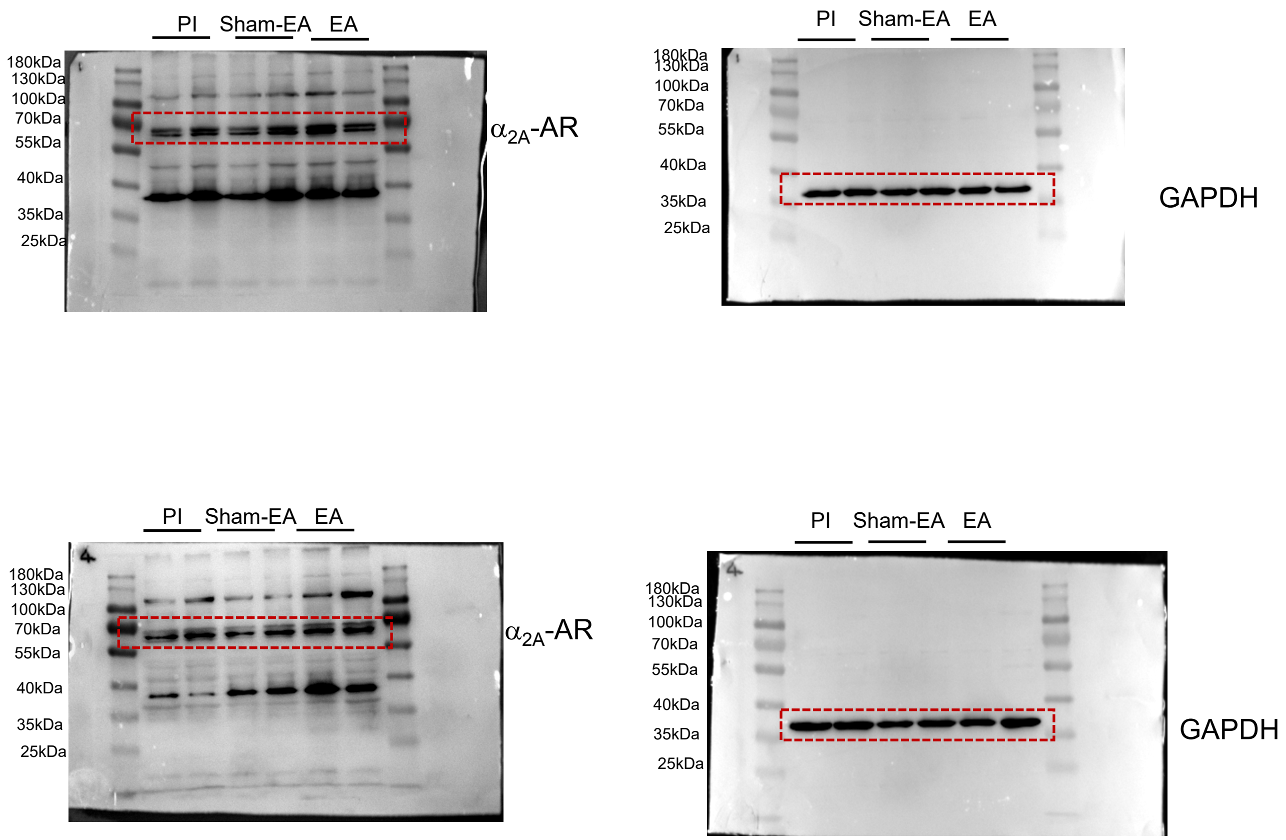

Fig 6

F

p-CaMKII

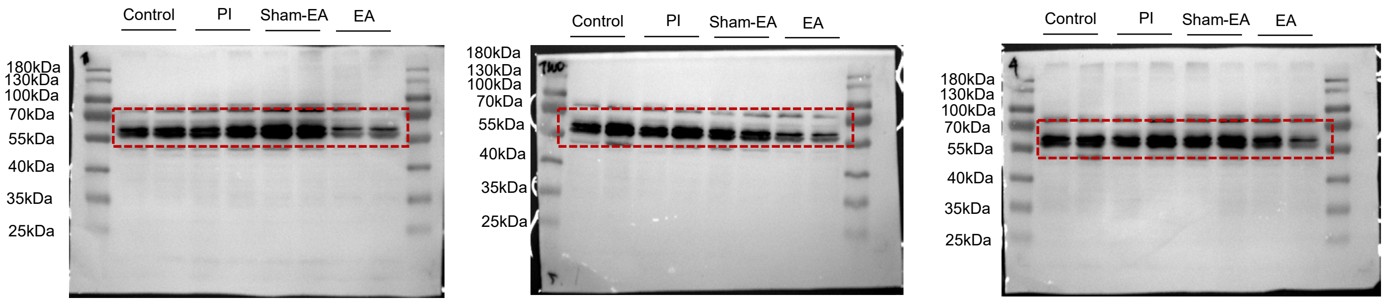

CaMKII

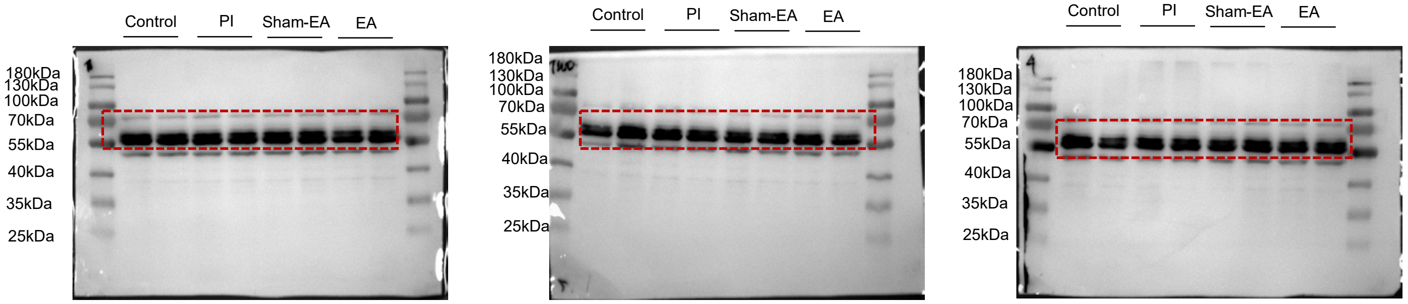

GAPDH

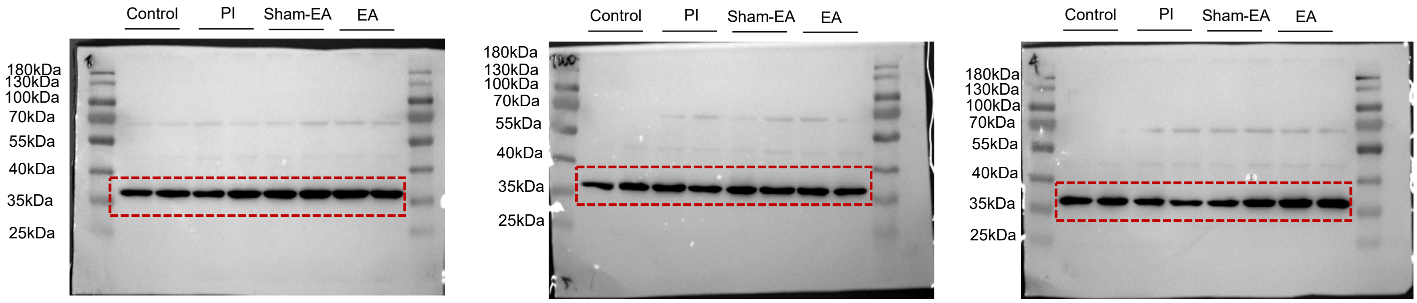

Fig 7B

p-CaMKII

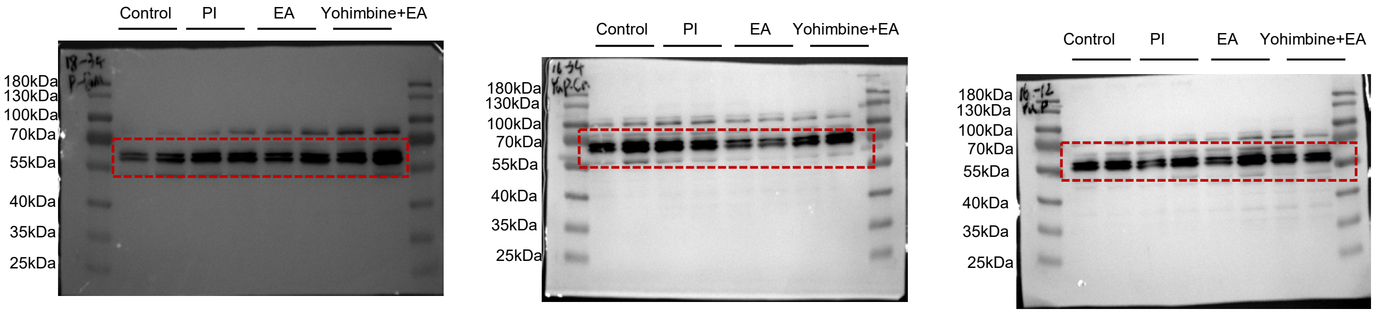

CaMKII

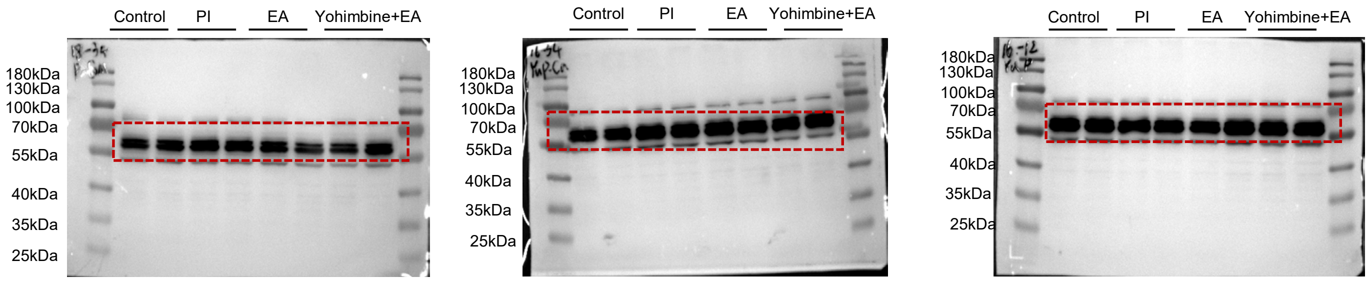

GAPDH

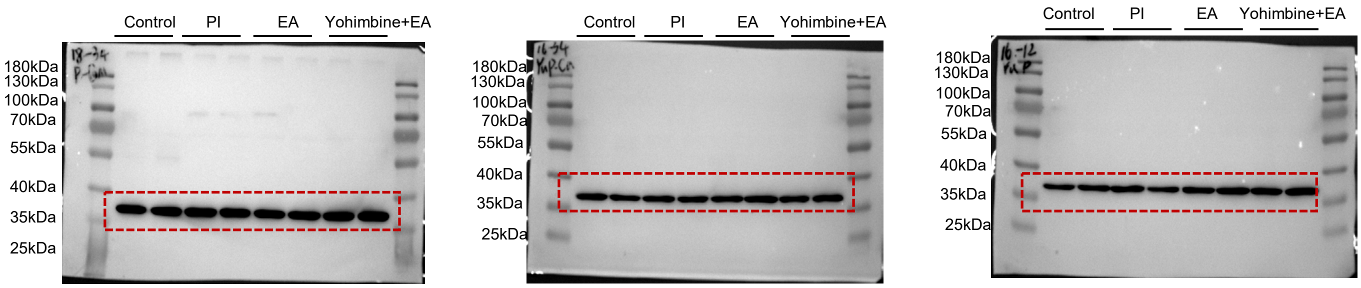

Fig S4 A

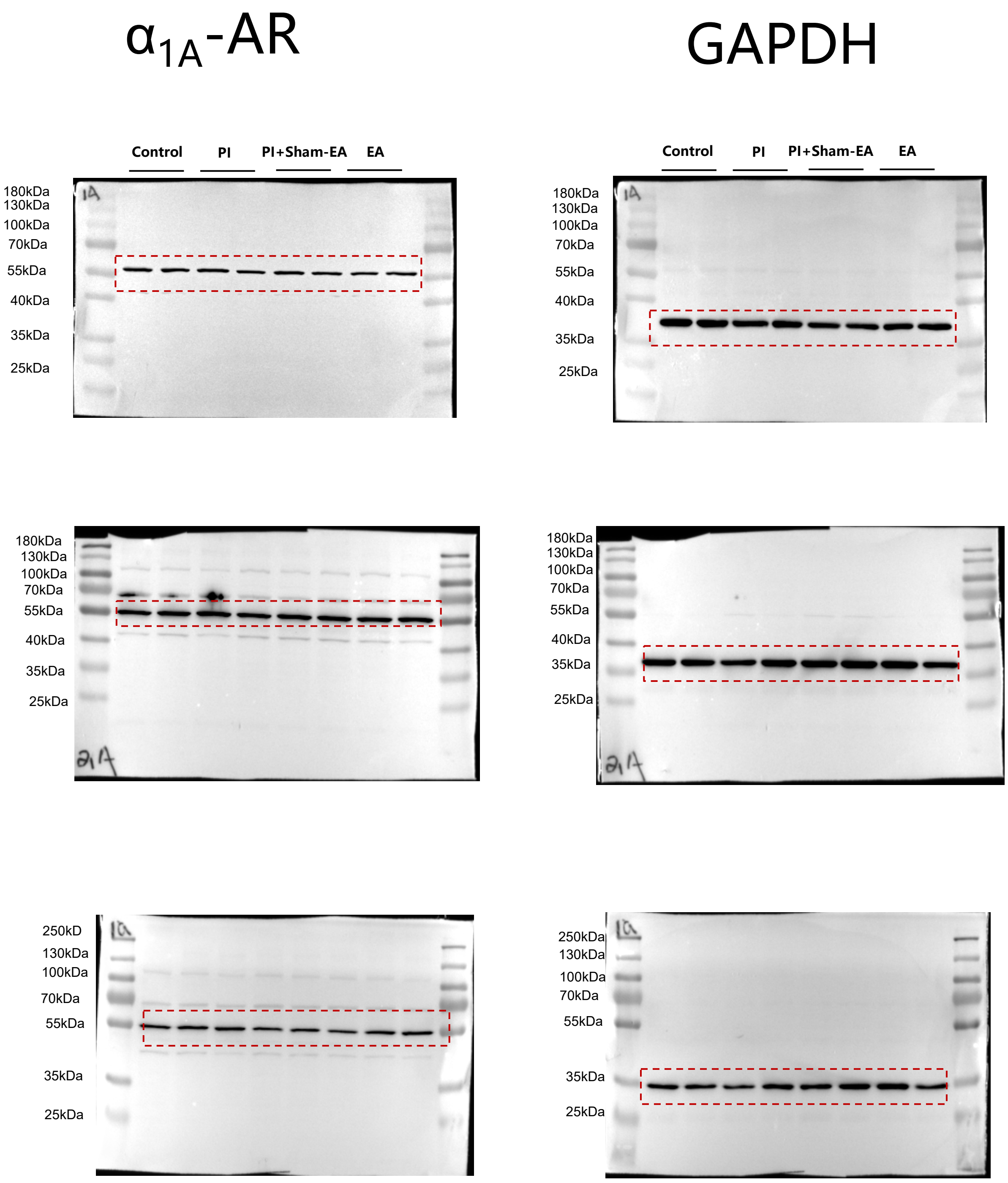

α1A-AR
GAPDH
Control
PI
PI+Sham-EA
EA
180kDa
130kDa
100kDa
70kDa
55kDa
40kDa
35kDa
25kDa
250kD
250kDa

Fig S4 B

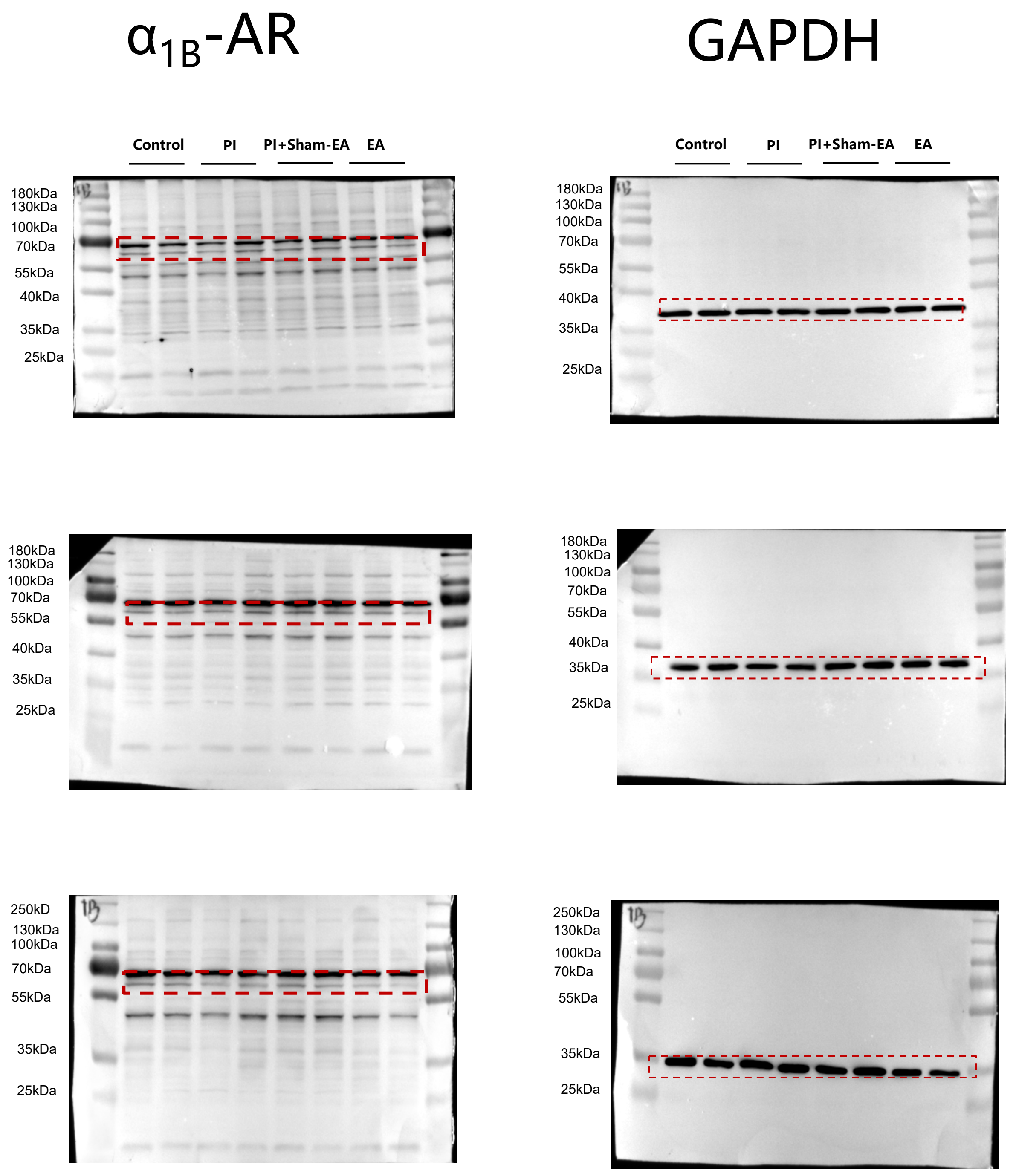
α1B-AR
GAPDH
Control
PI
PI+Sham-EA
EA
180kDa
130kDa
100kDa
70kDa
55kDa
40kDa
35kDa
25kDa
250kD
250kDa

Fig S4 C

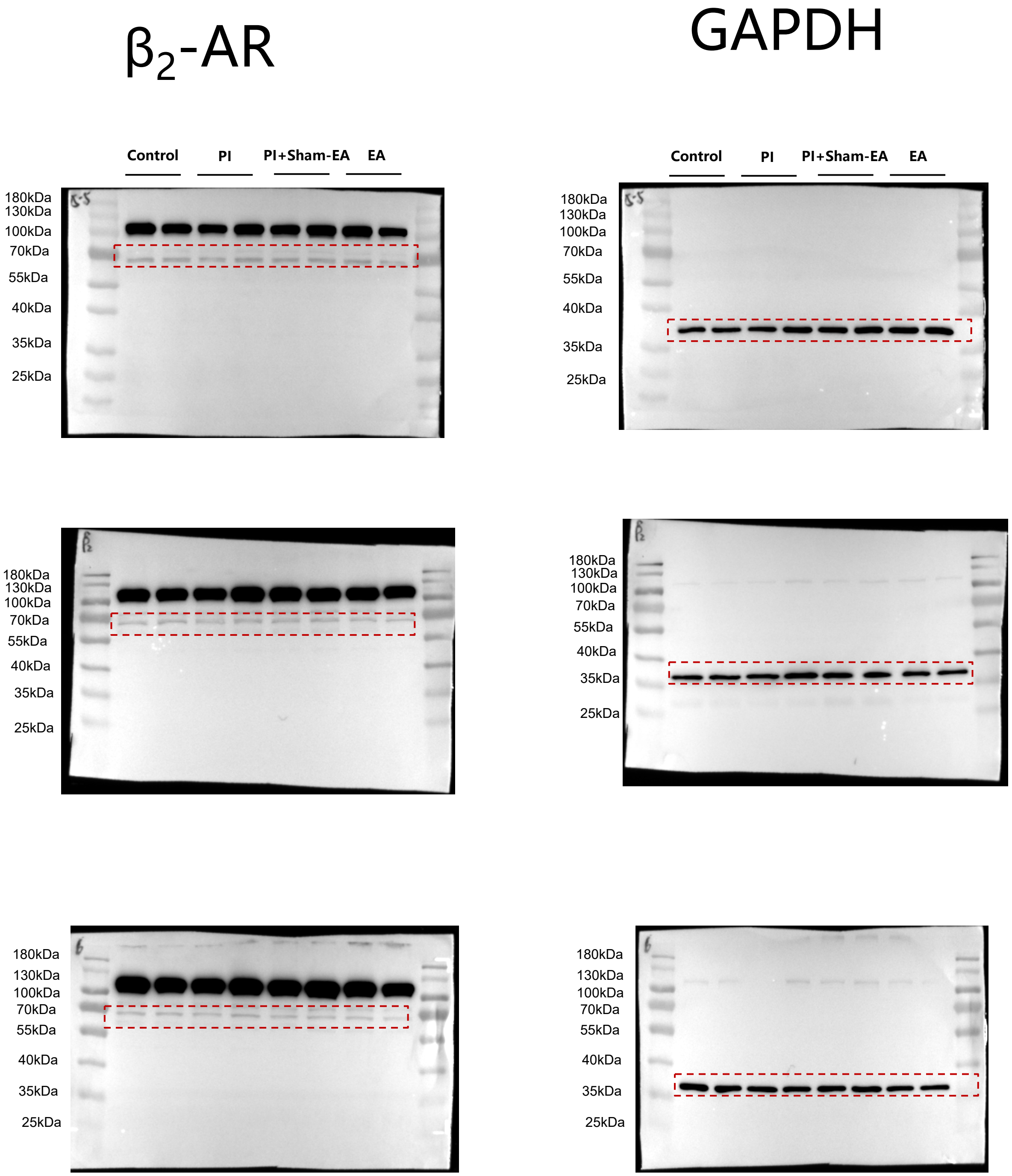

β2-AR
GAPDH
Control
PI
PI+Sham-EA
EA
180kDa
130kDa
100kDa
70kDa
55kDa
40kDa
35kDa
25kDa
